# Rapid Amplification of Cerebrospinal Fluid Pressure as a Possible Mechanism for Optic Nerve Sheath Bleeding in Infants With Nonaccidental Head Injury

**DOI:** 10.1167/iovs.65.12.9

**Published:** 2024-10-07

**Authors:** Peter S. Stewart, Bindi S. Brook, Oliver E. Jensen, Tamsin A. Spelman, Robert J. Whittaker, Moussa A. Zouache

**Affiliations:** 1 School of Mathematics and Statistics, University of Glasgow, Glasgow, United Kingdom; 2 School of Mathematical Sciences, University of Nottingham, Nottingham, United Kingdom; 3 Department of Mathematics, University of Manchester, Manchester, United Kingdom; 4 Sainsbury Laboratory, University of Cambridge, Cambridge, United Kingdom; 5 School of Mathematics, University of East Anglia, Norwich, United Kingdom; 6 John A. Moran Eye Center, Department of Ophthalmology & Visual Sciences, University of Utah, Salt Lake City, Utah, United States

**Keywords:** optic nerve hemorrhage, retinal hemorrhage, theoretical modelling, CSF flows

## Abstract

**Purpose:**

Subdural hemorrhage along the optic nerve (ON) is a histopathological indicator of abusive head trauma (AHT) in infants. We sought to determine if this bleeding could be caused by an abrupt increase in intracranial pressure transmitted to cerebrospinal fluid (CSF) at the optic foramen (OF).

**Methods:**

A theoretical model is developed to simulate the effect of a pressure perturbation of maximal amplitude *P* applied at the optic foramen for a short duration *T* on the CSF-filled ON subarachnoid space (ONSAS). The ONSAS is modelled as a fluid-filled channel with an elastic wall representing the flexible ONSAS–arachnoid/dura interface. A constitutive law describing the relationship between CSF pressure and ONSAS deformation is inferred from published measurements. CSF pressure profiles along the ONSAS are examined systematically over a broad range of *P* and *T*.

**Results:**

The pressure perturbation initiated at the OF produces a pressure wave that stretches the ONSAS. This wave propagates rapidly along the ONSAS toward the scleral end of the ON, where it is reflected back toward the brain. For sufficiently small *T* a shock wave with amplification up to six times larger than *P* over a timescale of tens of milliseconds is observed at the scleral end of the ON. Comparatively smaller amplifications are observed for slower perturbations.

**Conclusions:**

A sudden increase in CSF pressure in the cranial cavity can cause a rapid expansion of the ONSAS, which may lead to rupture of the bridging blood vessels. Our study predicts a plausible mechanism for subdural hemorrhage that occurs in abusive head trauma in infants.

Abusive head trauma (AHT), nonaccidental head injury, and shaken baby syndrome (SBS) are synonymous terms^1^ that have, variously over time, been given to the most common presentation of child abuse in which there is eye and intracranial pathology, with significant morbidity and mortality.^2^ Retinal hemorrhage, intracranial subdural hemorrhage, and encephalopathy are three postmortem clinical findings often considered to be hallmarks of AHT/SBS;^2^ although some controversies remain.^3^ Optic nerve sheath (ONS) hemorrhage, a common eye pathology finding in cases of fatal AHT where the ON is examined post mortem,^4^^–^^10^ has been proposed as a key finding in the diagnosis of the syndrome^5^^,^^7^ because it is more common in patients with AHT as compared with children having suffered accidental head injury.^5^^,^^7^^–^^9^ In addition to ONS hemorrhage and retinal hemorrhage, eye pathology findings of AHT include retinal detachment,^4^ circumferential retinal folds with macular schisis cavities (perimacular folds), peripapillary scleral haemorrhage,^9^ vitreous haemorrhage,^4^^,^^5^ choroidal haemorrhage,^4^ and orbital haemorrhage.^4^^–^^9^^,^^11^ However, these features are not entirely specific and differential diagnoses must be explored while excluding accidental injury and confounding conditions.^12^

Our current understanding of the mechanisms associated with indicators of AHT is largely empirical and often contentious. This is in part due to the fact that the chain of events leading to orbital and ocular injuries, such as ONS bleeding, after repeated shaking,^1^^,^^13^ has mainly had to be inferred from postmortem findings. ONS bleeding has been reported in other pathologies, including severe accidental trauma^14^^,^^15^ and in Terson's syndrome,^16^^,^^17^ which is characterized by vitreous or retinal hemorrhage associated with intracranial bleeding.^18^^,^^19^ Patients with Terson's syndrome are more than four times more likely to die as compared with patients presenting with subarachnoid hemorrhage only.^20^ ONS hemorrhage is occasionally seen in severe head injury in adults^21^^,^^22^ and has been linked to a sudden increase in intracranial pressure (ICP) in both Terson's syndrome^16^^,^^23^^,^^24^ and nonaccidental head injury in infants.^7^^,^^10^ Based on this finding, it is conceivable that the ONS bleeding observed in AHT is caused by an abrupt increase in ICP, even though the plausible time scales of ICP elevations in AHT, Terson's syndrome, and severe head injury in adults may differ.^25^^–^^28^

Although it is generally accepted that a sudden increase in ICP may cause hemorrhage of the ONS,^29^^–^^32^ the mechanisms causing this bleeding remain unsolved. Some have proposed that blood found postmortem within the ONS may flow directly from the cranial compartment through the connection between the intracranial cerebrospinal fluid (CSF) space surrounding the brain and the ON subarachnoid space (ONSAS) in the meningeal layer of the ONS^33^ (Fig. 1a). A sudden increase in the ICP may also cause an increase in intracranial venous pressure that is ultimately transmitted to the orbital veins, leading to a stasis in ocular blood drainage.^30^^,^^31^ Possible rotational and translational acceleration movements of the globe have been put forward in AHT/SBS.^7^ It has also been hypothesized based on postmortem findings that the transmission of ICP into the ONS occurs through the subarachnoid communication in the optic canal and not through blood vessels.^32^ This theory would explain why ONS hemorrhage is most commonly observed at the retrobulbar part of the ON.^4^^,^^5^^,^^7^

**Figure 1.**
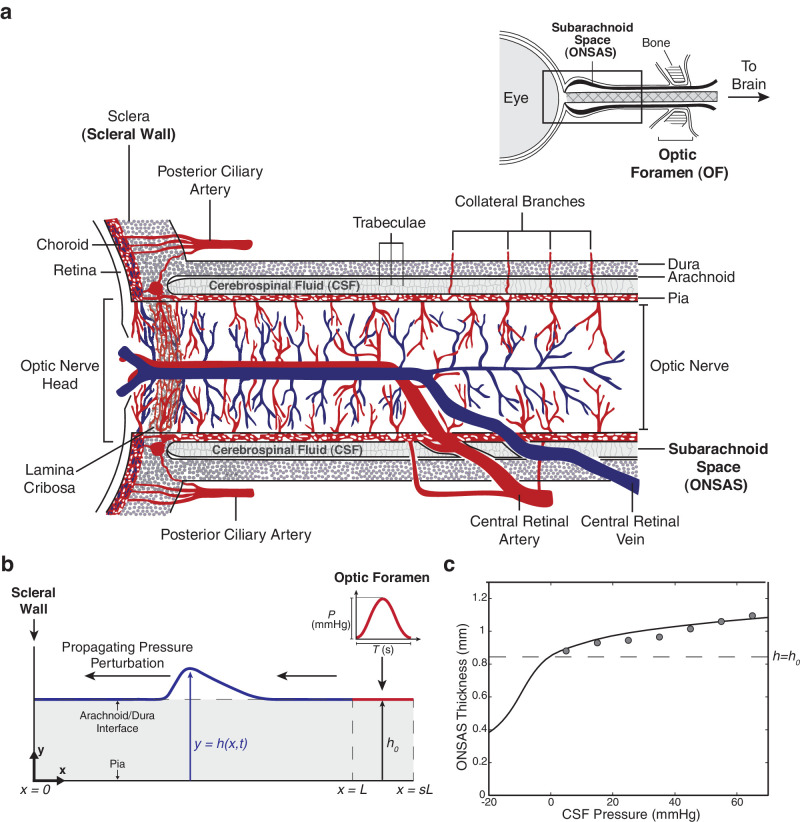
Geometry of the ON and ONSAS in human, with constitutive law describing the variation of the thickness of the ONSAS as a function of CSF pressure. (**a**) The ONSAS, which is filled with CSF, lies between the pia and arachnoid. The central retinal artery and central retinal vein are exposed to CSF pressure in the region where they cross the ONSAS, about 10 mm from the back of the eye. (**b**) Simplified geometry of the ONSAS used for the mathematical model. The model considers the flow of CSF along the ONSAS between the ON head and the OF. A sudden rise in ICP is modelled as a pulse in CSF pressure initiated at the distal end of the OF at *x* = *sL*. (**c**) Constitutive law describing the variation of the ONSAS thickness with CSF pressure (solid line, see Equation 1), determined by fitting ultrasound measurements of ON diameter during CSF infusion tests (*filled circles*).^34^^,^^35^

In this study we test the hypothesis that ONS bleeding can result from a sudden increase in ICP communicated to the CSF flowing into the ONSAS. We consider ICP perturbations over a range of time scales consistent with rapid head accelerations–decelerations.^25^^,^^26^ By using a mathematical model built on anatomical and physiological considerations, we examine the characteristics of this propagation and explore its consequences for the ONSAS and blood vessels bridging it. We show that an abrupt increase in ICP may be communicated to the CSF in the ONSAS. The resulting CSF pressure increase leads to a deformation of the ONSAS–arachnoid/dura interface that propagates along the ONSAS. This predicted change in ONSAS thickness may cause sudden, localized stretching or shearing, resulting in an increased vulnerability of blood vessels to rupture.

## Methods

### Geometry

We consider the ON and the elements enclosing it, which consist of the pia, ONSAS, arachnoid membrane and dura mater (see Fig. 1a and Table for a list of parameters). CSF flows through the ONSAS between the optic foramen (OF) and the region proximal to the ON head at the sclera (subsequently denoted as the scleral end of the ON). The ON and pia mater may be modelled as rigid structures impermeable to CSF that are fixed to the sclera. The dura and arachnoid membranes, which enclose the ONSAS, adhere together and are surrounded externally by soft fatty tissue. They are modelled as a deformable elastic sheet impermeable to CSF enclosing the ONSAS. We may, therefore, restrict the geometry considered to the ONSAS and model it as a fluid-filled channel with flexible walls representing the ONSAS-arachnoid/dura interface (see Fig. 1b, which extends the range of Fig. 1a to include the OF). The region of interest spans from the impermeable boundary at the scleral wall (*x = 0*) to the inlet of the CSF flow at the OF located at a distance *x = L* from the ON head at the sclera. The pressure perturbation is applied at the cranial side of the optic canal at the coordinate *x = sL*, with *s > 1*. Across the OF (*L* ≤ *x* ≤ *sL*) the governing equations of the system are assumed to be identical, but with a significantly increased wall stiffness to mimic the bone rather than the dural sheath. In the Supplementary Material, we demonstrate that the model predictions are remarkably unaffected by our modelling assumptions across the optic canal (see for instance the effect of rigid tapering of the channel walls through the bone^34^).

**Table.** List of Parameters Included in the Mathematical Model With Symbol and Typical Value or Range Considered

Parameter	Symbol	Value Used for Calculations	Source
Length of ONSAS	*L*	27 mm	Sheng et al.^35^
Baseline CSF pressure	*p*_0_	10 mm Hg	Sheng et al.^35^
Baseline width of ONSAS	*h*_0_	0.85 mm	Hansen et al. (2011)^36^
Resistance of arachnoid/dura to deformation	*K*	6 mm Hg	Estimated from Hansen et al. (2011)^36^
Density of CSF	ρ	1000 kg.m^−3^	Davson (1969)^37^
			Loth et al. (2001)^38^
Time interval of pressure increase	*T*	0.005 − 0.1 seconds	Empirically determined
Applied CSF pressure increase	*P*	5 − 100 mm Hg	Empirically determined
Distance from ON head to OF	*sL*	29.7 mm	Sheng et al.^35^

### Elastic Properties of the ON

To simulate the propagation of a pressure wave along the ONSAS and the deformation of this compartment as a result of it, the relationship between CSF pressure and ONSAS width must be known. Here, we seek to determine a standard macroscale law, which incorporates the contribution of all anatomical structures determining the width and compliance of the ONSAS, including arachnoid trabeculae, septa, and pillars.^34^^,^^39^ At baseline, we assume that the CSF pressure within the ONSAS, denoted *p_0_*, is uniform and that the ONSAS is of constant width, which is here denoted *h_0_*. We assume that the constitutive law for the deformation of the ONSAS is of the form

(1) $$\begin{array}{c} p-p_{0}=f\left(\frac{h}{h_{0}}\right)=K\left({\left(\frac{h}{h_{0}}\right)}^{m}-{\left(\frac{h}{h_{0}}\right)}^{-n}\right),\; \end{array}$$

where *p* is the CSF pressure, *h* is the thickness of the ONSAS, *K* is a stiffness parameter describing the resistance of the arachnoid/dura to deformation (equivalent to its elasticity, expressed in mm Hg), and *m* and *n* are non-negative exponents. We infer the constitutive law from published measurements of ON diameter as a function of CSF pressure, established in postmortem infusion tests^36^^,^^40^ consistently with other studies^41^; this constitutive law is plotted in Figure 1c. The ON diameter is imposed to be *D* = 3mm,^36^ which implies a baseline ONSAS thickness of *h*_0_ = 0.85 mm. The values of *m* and *n* are determined by first identifying feasible values and then performing least squares fitting for the one remaining unknown parameter *K*. By using the computational model developed by Brook et al. (1999),^42^ we set *m* = 10 and *n* = 3/2. This yields

(2) $$\begin{array}{c} K\approx 6.081\;\mathrm{mm}\;\mathrm{Hg}\approx 811.14\;\mathrm{Pa}.\; \end{array}$$

The fitted constitutive law obtained with these parameter values is plotted in Figure 1c. Further details of the constitutive law, the model assumptions and the parameter fitting can be found in the Supplementary Material.

### Model for the CSF

The CSF is modelled as an inviscid fluid of constant density ρ. We denote the position of the ONSAS elastic wall as *y* = *h*(*x*, *t*), where *t* is time (see Fig. 1b). Derivation of the equations describing CSF flow through the ONSAS is detailed in the Supplementary Material. These equations can be reduced to the well-known shallow water equations^43^ modified to account for the elastic constitutive law described in Equation 1. The derivation assumes that the typical width of the ONSAS is much smaller than its length and that the flow is moving sufficiently rapidly for viscous effects in the fluid to be effectively ignored. The variation in ONSAS thickness *h*(*x*, *t*) is described by

$$\begin{array}{ccc} \frac{\partial h}{\partial t}+\frac{\partial \left(\bar{u}h\right)}{\partial x} & \;= & 0,\\ \frac{\partial \bar{u}}{\partial t}+\bar{u}\frac{\partial \bar{u}}{\partial x} & \;= & -\frac{1}{\rho }\frac{\partial f}{\partial x}, \end{array}$$

where $\bar{u}=\bar{u}\left(x,t\right)$ is the CSF velocity averaged over the channel cross-section, and *f* is the constitutive function defined in Equation 1. This model has many similarities to that proposed for blood flow in the giraffe jugular vein by Brook et al.^42^^,^^44^

### Model for the Perturbation of the ICP

The ONSAS is initially considered to be of uniform width *h* = *h*_0_ and the CSF is assumed to be at rest with a uniform pressure *p* = *p*_0_. A sudden increase in the ICP is modelled as a pulse in CSF pressure initiated on the cranial side of the OF (illustrated in Fig. 1b). This pressure perturbation has the prescribed form

$$\begin{array}{ccc} p\left(sL,t\right) & \;= & p_{0}+P\sin^{2}\left(\frac{\pi t}{T}\right),\;\mathrm{for}\;0\leq t\leq T\\ p\left(sL,t\right) & \;= & p_{0},\;\mathrm{for}\;t>T \end{array}$$

and is, therefore, applied over the fixed time interval 0 ≤ *t* ≤ *T*. The perturbation pressure reaches a maximal pressure of *P* at *t* = *T*/2 and returns to baseline for *t* ≥ *T*. The governing equations are solved along the length of the domain 0 ≤ *x* ≤ *sL* using a numerical method based on the finite volume method.^42^ Note that the wall stiffness is assumed to be much greater through the OF (*L* ≤ *x* ≤ *sL*). Outputs of the calculations include the width of the ONSAS and the pressure and speed of the CSF at every point *x* for all times *t*. A specially adapted numerical scheme is necessary as these governing equations admit wave steepening which can lead to formation of a shock wave (a profile with infinite slope). The range of maximal pressures *P* and time intervals *T* considered were empirically determined based on physiological considerations and past experimental studies.^25^^–^^28^^,^^45^^–^^47^ The increase in pressure within the spinal cord caused by coughing has been estimated to be 50 to 70 mm Hg or more above the normal level.^48^ Although this increase may not be as severe at the ON, it indicates that CSF pressure perturbations associated with traumatic head injury may be very high. The frequency of head accelerations–decelerations applied in previous animal studies ranged approximately from 2 Hz^25^^,^^26^ to 15 Hz.^45^ To ensure that the solutions encompass physiologically realistic perturbations and gain valuable insights into mechanisms possibly leading to ON hemorrhage, we therefore consider a range of pressure pulses and time intervals, with 10 ≤ *P* ≤ 100 mm Hg and 0.005 seconds ≤ *T* ≤ 0.1 seconds.

## Results

### Propagation of the Pressure Wave along the ONSAS

**Figure 2.**
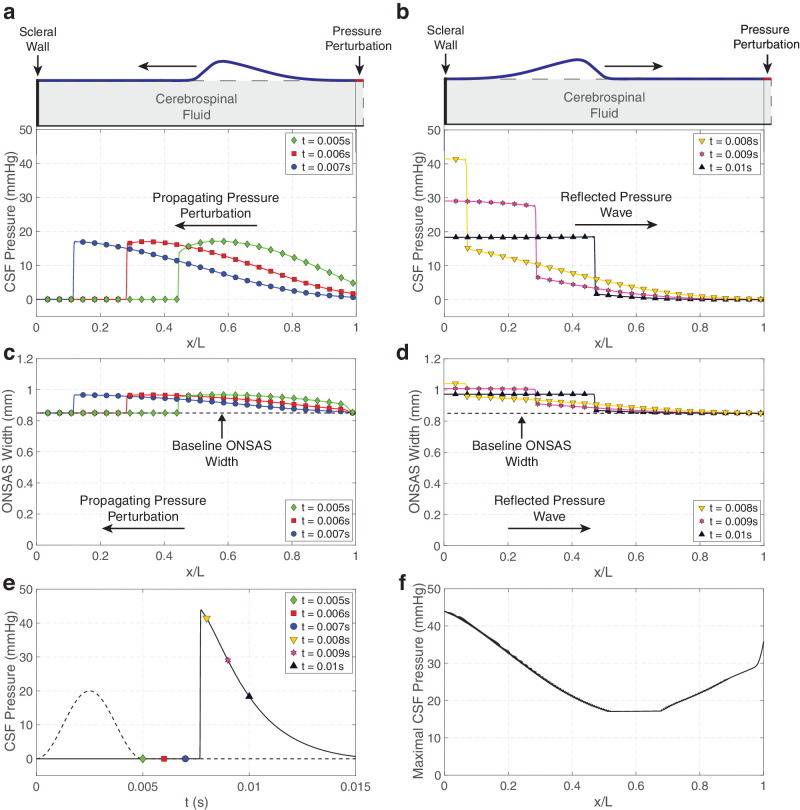
Characteristic variation of the ONSAS width and CSF pressure along this channel, shown for a perturbation of amplitude *P =* 20 mm Hg and time *T =* 0.005 seconds applied at the distal end of the ON at *x* = *sL*. (**a**) Pressure profiles, shown at *t* = 0.005 seconds, *t* = 0.006 seconds, and *t* = 0.007 seconds after perturbation, are characteristic of a shock wave. (**b**) Because the ONSAS is impermeable and fixed at the proximal end of the ON, the pressure wave, here shown at *t* = 0.008 seconds, *t* = 0.009 seconds, and *t* = 0.01 seconds after perturbation, are reflected back toward the OF. A two-fold amplification of the amplitude of the wave is observed. (**c**, **d**) The propagating increase in CSF pressure causes an expansion of the ONSAS. (**e**) Variation of the CSF pressure at the scleral wall as a function of time after perturbation (time trace). The perturbation is plotted as a dashed line. Markers and colors are consistent with the profiles shown in (**a**)–(**d**). A sharp amplification is observed at the scleral wall approximately *t* ≈ 0.0078 seconds after perturbation. (**f**) Variation of the maximal CSF pressure with distance from the scleral wall caused by the propagation and reflection of the pressure wave. The proximal end of the ON is the region with the largest increase in CSF pressure.

A rapid CSF pressure perturbation imposed at the OF propagates rapidly along the ONSAS toward the eye. As it propagates, this pressure wave deforms the interface between the ONSAS and the arachnoid/dura, leading to a local expansion of this space. Because the behavior associated with the deformation of the ONSAS is qualitatively similar to that associated with the changes in CSF pressure that cause it, we here describe the response to the imposed perturbation at the OF in terms of CSF pressure variation. The characteristics of this response are described in Figure 2 for a disturbance of *P* = 20 mm Hg applied over a time *T* = 0.005 seconds. A three-dimensional carpet plot showing the dynamics of this shock wave is provided in the Supplementary Material. The pressure wave displays a steepening leading edge (Fig. 2a), which eventually forms a shock wave (while the trailing wave is a rarefaction). Because the sclera is assumed impermeable to CSF, this shock wave is subsequently reflected back toward the brain (Fig. 2b) as a second shock wave of amplitude larger than the incident one. The local expansion of the ONSAS associated with the incident and reflected pressure waves propagates in a manner similar to CSF pressure waves (Figs. 2c and 2d). Reflection of the pressure shock wave causes an approximately two-fold increase in the CSF pressure at the scleral wall (*x* = 0), which gradually decreases as the wave propagates back to the OF. This sudden increase in CSF pressure can be seen in Figure 2e, which shows the temporal evolution of the prescribed CSF pressure perturbation (dashed line, imposed at *x = sL*) and the resulting CSF pressure at the scleral wall (solid line, measured at *x = 0*) at various time points after perturbation. For *P* = 20 mm Hg and *T* = 0.005 seconds, the maximal pressure at the scleral wall is approximately 2.4 times greater than the amplitude of the applied pressure perturbation. The spatial distribution of the pressure amplification showing the maximal CSF pressure measured at each point along the ONSAS is plotted in Figure 2f. Intervals where the CSF pressure exceeds the prescribed perturbation pressure are localized to the two ends of the ONSAS. The largest CSF pressure is observed in a short region immediately adjacent to the scleral wall, where amplification of the pressure wave occurs.

### Effect of Perturbation Time

**Figure 3.**
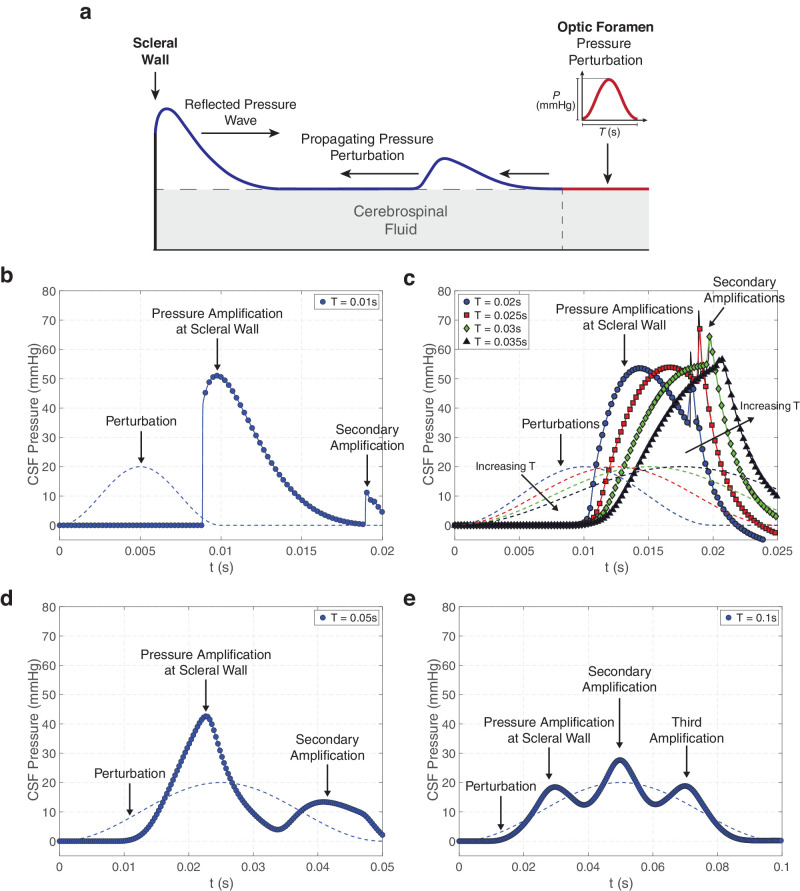
Time traces of CSF pressure, corresponding to the variation of CSF pressure at the scleral wall with time, following perturbations with duration *T* ranging from *T* = 0.01 seconds to *T* = 0.1 seconds and pressure amplitude *P* = 20 mm Hg. (**a**) Schematic showing the propagation of a pressure wave after perturbation at the OF and its reflection at the scleral wall. (**b**–**e**) Time-traces for (**b**) *T* = 0.01 seconds, (**c**) 0.02 ≤ *T* ≤ 0.035 seconds, (**d**) *T* = 0.05 seconds, and (**e**) *T* = 0.1 seconds. Profiles of the perturbation are plotted in dashed lines. When *T* > 0.01 seconds the maximal scleral wall pressure is determined by the propagation of a smooth pressure wave rather than by a shock wave (seen in Fig. 2 for *T* = 0.005 seconds). The reflected shock wave propagates rapidly back to the OF, where it is then again reflected toward the eye. This secondary arrival of the shock wave leads to a second, much less pronounced, increase in pressure at the scleral wall that occurs after the end of the perturbation. As the number of maxima increases with *T* the amplitude of the pressure at the scleral wall is reduced but remains larger than that of the initial perturbation.

**Figure 4.**
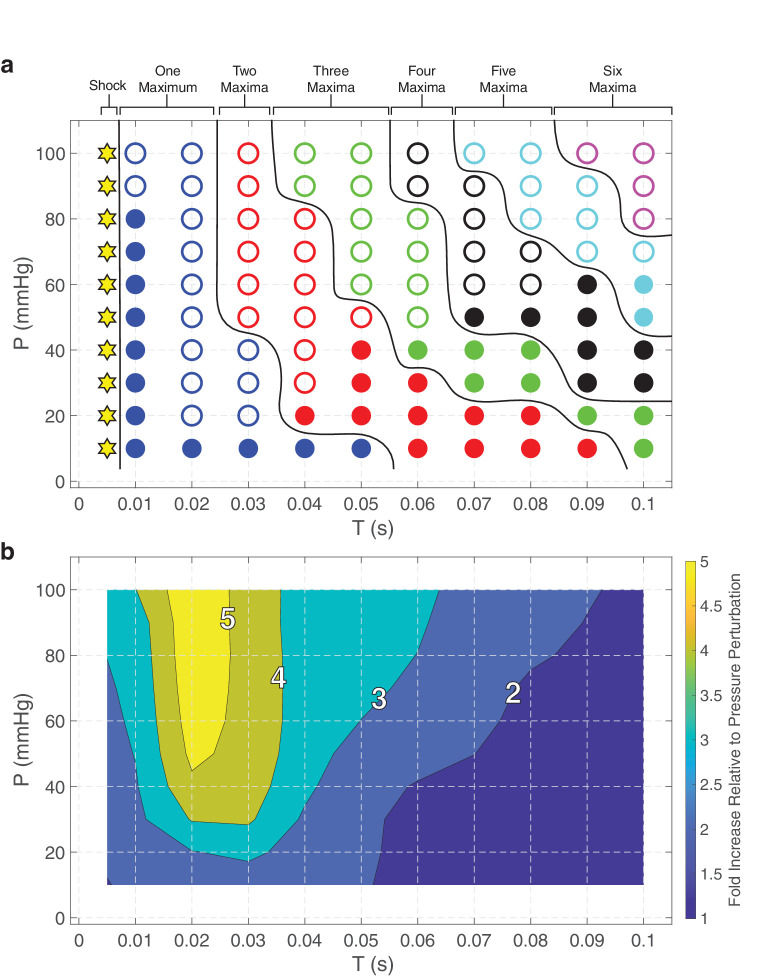
Regime diagrams describing the characteristics of the response to a pressure perturbation characterized by 0.005 seconds ≤ *T* ≤ 0.1 seconds and 5 mm Hg ≤ *P* ≤ 100 mm Hg applied at the OF. (**a**) Maxima in scleral wall pressure may be caused by the primary reflection of a shock wave (*stars*), propagation and reflection of a smooth pressure wave (*filled circles*) or as a result of secondary propagation/reflection of a shock wave combined with a smooth pressure wave (*open circles*). The number of maxima in pressure at the scleral wall gradually increases with both the perturbation time and the amplitude of the pressure perturbation. (**b**) The peak scleral pressure can be one to more than five times larger than the perturbation pressure *P*. The greatest increase is seen when a single scleral pressure peak is present.

The characteristic timescale *T* of the pressure perturbation is a key determinant of the variation of CSF pressure along the ONSAS and of its amplification at the scleral wall. To characterize this dependence, we analyzed time traces of CSF pressure that correspond with the temporal variation of CSF pressure at the scleral wall with time (see schematic in Fig. 3a), following comparatively longer perturbations, with *T* ranging from *T* = 0.01 seconds to *T* = 0.1 seconds while maintaining *P* = 20 mm Hg. The time trace of CSF pressure at the scleral wall for *T* = 0.01 seconds (Fig. 3b) is similar to that obtained for *T* = 0.005 seconds (see Fig. 2c). However, in this case, the maximal scleral pressure is reached at *t*  ≈  0.0095 seconds (Fig. 3b) after the arrival of the shock wave at the scleral wall (which takes place at *t*  ≈  0.008 seconds in Fig. 3b). For this larger value of *T*, the driving pressure continues to increase beyond the time the shock is formed. The CSF pressure behind the shock wave increases smoothly so that the maximal scleral pressure is associated with this smooth pressure wave (rather than the arrival of the shock wave). The reflected shock wave is still present and propagates rapidly back toward the OF, where it is then again reflected toward the eye owing to the significantly increased wall stiffness across the OF (*L* ≤ *x* ≤ *sL*). This secondary arrival of a shock wave leads to a second, much less pronounced, increase in pressure at the scleral wall that occurs after the end of the perturbation (at *t*  ≈  0.018 seconds in Fig. 3b). Similar behavior is observed as *T* is increased, where eventually the propagating pressure wave does not steepen sufficiently to form a shock before reaching the scleral wall, so that the initial increase in scleral pressure is smooth (Fig. 3c). In some cases, the reflected (smooth) pressure wave steepens to form a shock wave, which propagates back toward the OF, where it is re-reflected back toward the sclera. This secondary shock wave reaches the scleral wall after the initial peak, creating a second abrupt increase in CSF pressure in this region (seen at *t* > 0.018 seconds in Fig. 3c). This abrupt increase in CSF pressure can in some cases be more than five times the imposed perturbation amplitude (see Fig. 4). The amplitude of this secondary shock reduces as *T* increases (for instance *T* = 0.03 seconds or *T* = 0.035 seconds in Fig. 3c). We also observed that the number of complete propagation/reflection cycles for the pressure wave increases with *T*. A three-dimensional carpet plot showing the propagation of these primary and secondary waves for *T* = 0.02 seconds is provided in online Supplementary Material. For *T* = 0.05 seconds, the time trace of scleral pressure exhibits two smooth maxima (Fig. 3d), whereas for *T* = 0.1 seconds the time trace exhibits three smooth peaks (Fig. 3e). As the number of maxima increases the maximal amplitude of the pressure at the scleral wall is decreased, but remains slightly larger than that of the initial perturbation *P*. A three-dimensional carpet plot showing the back-and-forth propagation of the pressure wave along the ONSAS for *T* = 0.05 seconds is provided in the Supplementary Material.

### Systematic Parameter Analysis

The response to a perturbation applied at the OF takes distinct forms depending on the time and amplitude of the perturbation. To characterize these dynamics, we performed a systematic analysis of the response to perturbation across a broad range of values of *T* and *P*, with 0.005 seconds ≤ *T* ≤ 0.1 seconds and 10 mm Hg ≤ *P* ≤ 100 mm Hg. Behaviours predicted by the model are shown in Figure 4, illustrating both the source of the maximal scleral pressure (Fig. 4a) and the corresponding maximal amplification of the scleral pressure compared with the input (Fig. 4b). Over the range of parameters considered, the maxima in scleral wall pressure may be caused by either the primary reflection of a shock wave (stars in Fig. 4a, similar to Fig. 3b), propagation and reflection of a smooth pressure wave (filled circles in Fig. 4a, similar to Figs. 3d and 3e) or as a result of secondary propagation/reflection of a shock wave combined with a smooth pressure wave (open circles in Fig. 4a, similar to Fig. 3b). Overall, a maximal scleral pressure owing to shock wave propagation alone is restricted to very short perturbation times (*T* < 0.01 seconds) (Fig. 4a). In addition, the number of pressure peaks gradually increases with both the perturbation time and the amplitude of the pressure perturbation (Fig. 4a). The influence of secondary shock wave propagation/reflection becomes more pronounced as the driving pressure amplitude increases (Fig. 4a). Simulations indicate that the peak scleral pressure can be almost six times larger than the perturbation pressure *P* (Fig. 4b). This large increase occurs when *T* is such that secondary propagation and reflection of a shock wave superimposed on the primary wave propagation leads to a dramatic increase in scleral pressure (behavior similar to that associated with *T* = 0.025 seconds in Fig. 3c).

## Discussion

A sudden increase in the CSF pressure initiated at the cranial end of the ON can drive a pressure wave that causes a local expansion of the ONSAS. This pressure wave propagates rapidly toward the eye and is reflected at the scleral end of the ON. As a result of this reflection the pressure wave is significantly amplified in the region adjacent to the ON head at the sclera (Fig. 2). Upon reaching the OF this reflected wave is re-reflected back toward the eye, creating a secondary peak in CSF pressure at the sclera (Figs. 2 and 3). For sufficiently long perturbation times this back-and-forth propagation can lead to multiple local maxima in CSF pressure and ONSAS width over the timescale of pressure perturbation (Fig. 3). The time and maximal amplitude of the pressure perturbation are key determinants of the variation of the CSF pressure along the ON. A thorough parameter analysis indicates that the amplification seen at the scleral end of the ON can be up to almost six times larger than the associated CSF pressure perturbation over a timescale of tens of milliseconds (Fig. 4 and Supplementary Material).

This analysis introduces a novel plausible mechanism for ON damage and associated hemorrhage seen in AHT and more generally in traumatic brain injury. The severe elevation in ICP caused by AHT is communicated to the CSF in the ONSAS. The resulting increase in CSF pressure triggers a propagating CSF tsunami along the ONSAS, which steepens to form a shock. Reflection of this wave at the scleral end of the ON results in a transient pressure increase. Blood vessels bridging the ONSAS are exposed to this large increase in CSF pressure (and accompanying abrupt increase in ONSAS width) and are hence vulnerable to stretching, shearing, and rupture, causing bleeding. The central retinal vein, which is generally more distensible than the central retinal artery, may be particularly vulnerable to compression as it traverses the ONSAS. Central retinal vein occlusion, accompanied by the reflex arterial pressure elevation owing to an increased ICP, may then lead to the retinal pathology.^32^ Our model predicts that the highest increase in CSF pressure after a severe elevation in ICP is localized to the scleral end of the ON. This finding may explain why ONS hemorrhage is most commonly found at the retrobulbar part of the ON attached to the eye.^4^^,^^5^^,^^7^

The nature of the perturbation caused to ICP or CSF pressure by a traumatic head injury is difficult to determine experimentally. The ensuing formation of CSF pressure pulses (and associated expansion of the ONSAS) is plausible as they are known to occur even during simple cough.^48^ The assumption of a pressure wave propagating in CSF has been proposed as a possible mechanism for syringomyelia,^49^^,^^50^ where the steepening pressure (shock) wave is reflected by an occlusion in the spinal column. A key finding of our study lies in the dependency of the response to an increase in CSF pressure on the timescale *T* of the pressure perturbation. For rapid perturbations the system forms a rapidly propagating shock wave, which is significantly amplified by reflection at the sclera. However, for slower perturbations, the system instead exhibits almost no amplification. The model predicts that the largest increase in CSF pressure occurs for large pressure perturbations (*P* > 40 mm Hg) applied over very short timescales (*T* ≈ 0.02 seconds). This maximum coincides with the greatest likelihood of causing damage to the ONS. It is plausible that consecutive, high-frequency pressure perturbations, such as those likely to occur in AHT/SBS, may generate multiple pressure waves along the ONSAS. Amplifications that are much larger than each individual pressure perturbation may then arise (see Supplementary Fig. S5 in the Supplementary Material), dramatically increasing the risk for damage to the ONS. This finding is significant, as it may explain why ONS bleeding is less commonly observed in accidental head injury in infants, where trauma is less likely to involve repeated acceleration-decelerations of the head.^1^^,^^13^ Therefore, our study is consistent with the use of ONS bleeding as a marker differentiating accidental from nonaccidental head injury in infants. However, it should be noted that more work is necessary to characterize perturbations to CSF pressure associated with accidental head injury. The parameter analysis highlights the existence of a broad spectrum of responses depending on *P* and *T* (Fig. 4). We show in the Supplementary Information that scleral pressure amplification is also evident with other forms of acute pressure perturbation, such as a rapidly oscillating ICP (which might mimic a traumatic shaking event) or a persistently increased ICP (which might arise owing to brain oedema in the aftermath of a traumatic injury). The variability in susceptibility to ONS damage that the spectrum of responses reflects could explain ongoing controversies regarding the mechanisms driving ON hemorrhage in AHT/SBS.^51^

Additional indicators of AHT may be linked to the increase in CSF pressure in the ON following a sudden rise in ICP. The central retinal vein may be particularly vulnerable to compression as it traverses the ONSAS space. The rapid pressure increase in the ONSAS may be transmitted externally to the central retinal artery and vein and possibly to the retinal circulation. This scenario is consistent with computational modelling of blood flow along the central retinal vessels through the ON, which has demonstrated how abrupt CSF pressure changes can induce the propagation of pressure waves in the retinal circulation,^52^ under both normal conditions as a model of the retinal venous pulse^53^^,^^54^ and in response to an abrupt increase in the external pressure in the ONSAS.^52^ Propagation of these waves through the bifurcating network of retinal vessels could lead to blood vessel rupture and subsequent retinal hemorrhage, in line with the pressure increase hypothesis.^23^^,^^55^^,^^56^ This outcome is highly dependent on the timescale *T* of the prescribed pressure perturbation, as a slowly increasing ICP (caused by a tumor for instance) is not typically associated with retinal haemorrhage.^55^^,^^57^^–^^59^

Our model is subject to a number of limitations, particularly neglecting viscous effects in the CSF and modelling the elasticity of the dura mater/arachnoid membrane in a relatively simple manner. The elastic stiffness of this interface was inferred from data collected in adults and therefore neglects the additional pliability of intracranial structures when fontanelles have not yet fused in infants. Anatomical variations in the complex system of trabeculae, pillars and septa that partly maintain the structural integrity of the ONSAS along the ON^34^^,^^39^ may influence the propagation of CSF pressure waves and the deformation of this chamber in ways that have not been modelled. However, the theoretical model incorporates all the key considerations necessary to describe wave propagation along the ONS at the macro scale. Predictions from the model are remarkably robust to variations in the governing parameters (see the Supplementary Information). Our model therefore provides a valuable tool to test clinical hypotheses in a system where experimental data are difficult to generate.

## Supplementary Material

Supplement 1
