# Supplementary material for: Rapid Amplification of Cerebrospinal Fluid Pressure as a Possible Mechanism for Optic Nerve Sheath Bleeding in Infants With Nonaccidental Head Injury
Source: Invest Ophthalmol Vis Sci. 2024 Oct 7;65(12):9. doi: 10.1167/iovs.65.12.9 (PMC11463713; doi:10.1167/iovs.65.12.9)
Supplement: Supplement 1 [file iovs-65-12-9_s001.pdf]

Rapid amplification of cerebrospinal fluid pressure as a possible mechanism for optic nerve sheath bleeding in infants with non-accidental head injury: Supplementary material

Peter S. Stewart, Bindi S. Brook, Oliver E. Jensen, Tamsin A. Spelman, Robert J. Whittaker & Moussa A. Zouache

June 26, 2024

In this supplementary material we provide more details on our mathematical model for the flow of CSF along the optic nerve subarachnoid space (ONSAS) in response to an abrupt increase in intracranial pressure (mimicking a traumatic brain injury). Firstly, we characterise the deformation and elastic response of the optic nerve (ON) as a function of the local CSF pressure by interpreting measurements obtained from post-mortem infusion testing of human optic nerve tissue [1] (Sec. 1). Secondly, we construct a theoretical model for the flow of CSF along the ONSAS (Sec. 2) to predict the amplification in CSF pressure at the sclera following a large amplitude pressure perturbation at the optic foramen, using both analytical (Sec. 3) and numerical methods (Sec. 4). We end with a summary of the model predictions spanning all the different approaches (Sec. 5).

1 Constitutive model for the dura mater

Hansen *et al.* (2011) [1] (see also earlier work [2]) used ultrasound to measure the outer diameter of the optic nerve sheath (denoted here as D_{ons}) as a function of local CSF pressure in the ONSAS during infusion tests in post-mortem human optic nerve tissue. By assuming a nerve fibre diameter $D_{on} = 3$mm [3], we estimate the corresponding thickness of the ONSAS, denoted h, for a given CSF pressure. The estimated ONSAS thickness is plotted as a function of CSF pressure (filled circles on Fig. 1c in the main text). To close the mathematical model constructed below, we use this data to estimate a constitutive relationship between the CSF pressure (p) and the ONSAS thickness (h) relative to the baseline thickness (h_0). Such constitutive laws have been widely used in building models of blood flow in large arteries and veins (*e.g.* [4–7]). In particular, we assume that our constitutive law for the dural sheath takes the nonlinear form

$$p - p_0 = f(h/h_0) = K\left((h/h_0)^m - (h/h_0)^{-n}\right), \tag{S1a}$$

where K is a stiffness parameter measuring the resistance of the dural sheath to expansion or compression, p_0 is the corresponding baseline CSF pressure and m and n are non-negative exponents. When $h = h_0$, Eq. (S1) indicates that $p = p_0$, as expected. The data of Hansen *et al.* (2011) [1] with $D_{on} = 3$mm implies a baseline ONSAS thickness of $h_0 = 0.85$mm. Our approach is to choose m and n as feasible values and then perform least squares fitting for the one remaining unknown parameter K. In order to make use of the sophisticated computational model developed by Brook *et al.* (1999) [5] we set $m = 10$ and $n = 3/2$, where we then obtain

$$K \approx 6.08082\,\text{mmHg} \approx 811.14\,\text{Pa}, \tag{S1b}$$

shown as the solid line on Fig. 1c in the main text.

2 The model

We consider a two-dimensional cross-section through the ONSAS oriented parallel to the axis of the ON. We model the ON and pia mater as a rigid, flat, impermeable surface forming one wall of the channel, as sketched in Fig. 1b in the main text. The coordinates x and y are used respectively to describe distances in the directions along and perpendicular to the ON. Time is denoted t. In this simplified setup the sclera is located at $x = 0$, the inlet to the ONSAS (*i.e.* the distal end of the optic foramen) located at $x = L$ and the proximal end of the optic foramen located at $x = sL$ ($s > 1$); the pressure perturbation is applied at the inlet to the optic foramen ($x = sL$), mimicking a traumatic injury to the brain.

We model the dura mater and arachnoid membrane as an elastic membrane with a constitutive law linking the local CSF pressure to the local ONSAS width in the form of Eq. (S1). We denote the position of the dura as $y = h(x, t)$. We adopt two complimentary approaches to model the corresponding flow through the optic foramen ($L \leq x \leq sL$), described in Sec. 2.1 and Sec. 2.2 below.

The CSF is assumed to be an ideal fluid with constant density ρ. Denoting the two-dimensional fluid velocity field in the ONSAS as $\mathbf{u} = (u, v)$ and the CSF pressure as p, the flow is governed by the incompressible Euler equations [8]

$$\nabla \cdot \mathbf{u} = 0, \quad \mathbf{u}_t + \mathbf{u} \cdot \nabla \mathbf{u} = -\frac{1}{\rho} \nabla p, \tag{S2a}$$

for $0 \leq x \leq sL$ and $0 \leq y \leq h(x, t)$. The inviscid flow is subject to the no-penetration condition $v = 0$ on the ON at $y = 0$, as well as kinematic and continuity of normal stress conditions on the dura mater on $y = h(x, t)$,

$$v = h_t + u h_x, \qquad p - p_0 = f(h/h_0), \tag{S2b}$$

where $f(h/h_0)$ is the fitted constitutive law (S1).

Taking the cross-sectional average of (S2) leads to the classical (inviscid) shallow water equations [8]

$$h_t + (\overline{u}h)_x = 0, \quad \overline{u}_t + \overline{u}\overline{u}_x = -\frac{1}{\rho} p_x. \tag{S3}$$

Substituting the fluid pressure from the constitutive law (S1), we obtain a closed system involving only two dependent variables, the channel width h and the fluid velocity $\overline{u}$, in the form

$$h_t + (\overline{u}h)_x = 0, \quad \overline{u}_t + \overline{u}\overline{u}_x = -\frac{1}{\rho} f_x(h/h_0), \tag{S4a}$$

where $\overline{u} = \overline{u}(x, t)$ is the cross-sectionally averaged fluid velocity along the channel.

Initially, the ONSAS is considered to be of uniform width $h = h_0$, while the CSF is assumed to be at rest with uniform pressure $p = p_0$. To mimic an injury we consider a prescribed (abrupt) rise in CSF pressure of amplitude P (mmHg) over a timescale T (s) applied at the proximal end of the optic foramen ($x = sL$), which takes the form

$$p(sL, t) = p_0 + P \sin^2(\pi t/T), \qquad (0 \leq t \leq T), \tag{S4b}$$

$$p(sL, t) = p_0, \qquad (t > T). \tag{S4c}$$

In simulations discussed in the main paper we choose T in the range $T = 0.005$s to $T = 0.1$s. This pressure perturbation generates a pressure pulse which propagates along the ONSAS towards the sclera.

To close the model we assume the sclera ($x = 0$) is impermeable and impose boundary conditions of no flow in the form

$$\overline{u}(0, t) = 0. \tag{S4d}$$

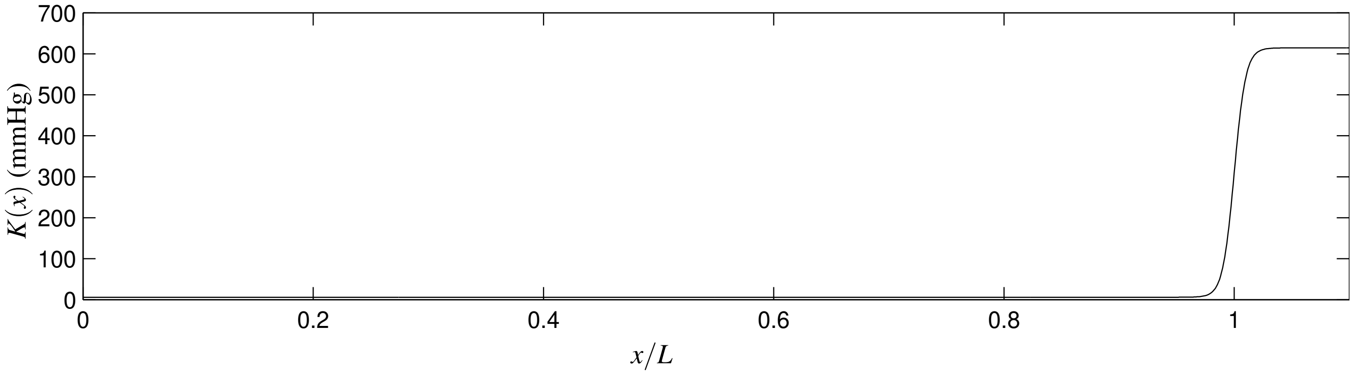

Figure S1: The spatially varying elastic stiffness used in the model without membrane tension.

In simulations of the model we compute the amplitude of the scleral pressure reflection as the ratio of the maximal scleral pressure (over a time interval of twice the perturbation timescale, T) to the prescribed inlet pressure, in the form

$$R \equiv \frac{\max_t(p(0,t))}{P}. \tag{S5}$$

This quantity is plotted in the main text as a function of the pressure perturbation amplitude and the time interval of pressure perturbation (Fig. 4b).

To close the model we use two approaches. In the first we model the optic foramen as a flexible-walled channel with the same constitutive law as the dura mater, but substantially increase the elastic stiffness to mimick the relatively rigid orbit (Sec. 2.1), while in the second we modify the constitutive model for the dura mater to include an axial tension (which increases the order of the equations) and treat the section of the channel through the optic foramen as entirely rigid (Sec. 2.2). We show in Sec. 5 below that these two methods produce both qualitatively and quantitatively similar results, and serve to validate our analytical and numerical methods.

2.1 Model 1: no membrane tension

In the absence of membrane tension we mimic the stiffer region through the optic foramen by modifying the constitutive law (S1). In this case, we choose

$$f(\alpha) = \overline{K}(x)\left(\alpha^{10} - \alpha^{-3/2}\right), \qquad (0 \le x \le sL), \tag{S6a}$$

where the stiffness distribution is chosen as

$$\overline{K}(x) = K\left(1 - \tfrac{1}{2}a\tanh(b(x-L)/L)\right), \qquad (0 \le x \le sL); \tag{S6b}$$

in simulations we choose the parameters $a = 100$ and $b = 100$. This stiffness function is plotted in Fig. S1. In this case disturbances propagate along the ONSAS at the dimensional wave-speed $c(h)$ (non-constant, in general) satisfying

$$c^2 = \frac{h}{\rho}\frac{\mathrm{d}f}{\mathrm{d}h} = \frac{h}{\rho h_0}\frac{\mathrm{d}f}{\mathrm{d}\alpha}, \qquad (0 \le x \le sL). \tag{S7}$$

Simulations of this model (S4,S6) across the domain $0 \le x \le sL$ are described in the main text.

2.2 Model 2: inclusion of axial tension

In order to validate the approach taken in Sec. 2.1, we also perform simulations where the constitutive model for the ONSAS width is modified to include an axial wall tension. In this case the order of the

equations is increased from two to four, allowing us to model the stiffer region through the optic foramen as entirely rigid. In this case we modify the constitutive law (S1) for the compliant section in the form

$$f(\alpha) = K\left(\alpha^{10} - \alpha^{-3/2}\right) - T_0 h_0 \alpha_{xx}, \qquad (0 \le x \le L), \tag{S8}$$

where T_0 is the axial membrane tension coefficient (assumed constant); to the best of our knowledge it is not possible to estimate this parameter *in vivo*. In simulations below we choose $\mathcal{T} = T_0/(h_0 K) = 10^{-4}$, ensuring the simulations are qualitatively similar to the case without membrane tension (described in main text).

Through the optic canal ($L \le x \le sL$) we assume that the channel is entirely rigid, with constant width h_0. Applying the governing equations (S3), we obtain

$$\overline{u}_x = 0, \qquad \overline{u}_t = -\frac{1}{\rho}p_x, \qquad (L \le x \le sL). \tag{S9}$$

In this case we can solve for the flow across the rigid segment analytically, to compute a boundary condition imposed at the end of the compliant segment ($x = L$) in form

$$p(L,t) - \rho(s-1)L\overline{u}_t(L,t) = p(sL,t), \tag{S10a}$$

where the term on the right-hand-side is the prescribed function (given by Eqs. (S4b) and (S4c)). In the presence of membrane tension the order of the PDE system (Eq. S4) is increased by two due to the second derivative in (Eq. S8), and so two further boundary conditions are required. In this case we impose the additional conditions

$$h_x(0,t) = 0, \qquad h(L,t) = h_0. \tag{S10b}$$

At the inlet to the ONSAS (the distal end of the optic foramen) the ONSAS width is fixed to the width of the rigid channel, whereas the slope condition on the dural sheath at the sclera is chosen for numerical convenience.

Simulations of this model with membrane tension (Eqs. S4, S8, S10) across the domain $0 \le x \le L$ are described in Sec. 5.2 below.

3 Analytical solutions for propagating shock waves along the ONSAS

In numerical simulations described in the main text we elucidate how the applied pressure disturbance (Eq. S4b) can generate a propagating shock wave along the ONSAS when the perturbation timescale is sufficiently short ($T < 0.01$s). By assuming this pressure rise instantaneously generates a propagating shock wave of the same amplitude, we can analytically describe the propagation and reflection of this shock wave using the Rankine–Hugoniot conditions.

Initially the shock wave advances along the ONSAS toward the sclera with speed V_{01}. Ahead of the shock the flow is undisturbed and so $h = h_0$, $p = p_0$ and $\overline{u} = 0$ (from the assumption of an impermeable sclera); behind the shock we denote $h = h_1$, $p = p_1 = p_0 + f(h_1/h_0)$ and $\overline{u} = u_1$. Mass and momentum are conserved across the shock, as expressed by the Rankine–Hugoniot conditions [8] in the form

$$(u_1 - V_{01})h_1 = -V_{01}h_0, \tag{S11a}$$

$$\tfrac{1}{2}\rho(u_1 - V_{01})^2 + p_1 = \tfrac{1}{2}\rho(-V_{01})^2 + p_0. \tag{S11b}$$

At time $t = t_s = L/V_{01}$, the shock hits the sclera at $x = 0$, causing a reflected shock travelling with speed V_{12} back towards the optic foramen. Ahead of the reflected shock $h = h_1$, $p = p_1 = p_0 + f(h_1/h_0)$ and $\overline{u} = u_1$, while behind the reflected shock we denote $h = h_2$, $p = p_2 = p_0 + f(h_2/h_0)$ and $\overline{u} = u_2 = 0$ (again from the assumption of an impermeable sclera). In this case, the Rankine–Hugoniot conditions give

$$(u_1 - V_{12})h_1 = -V_{12}h_2, \tag{S12a}$$

$$\tfrac{1}{2}\rho(u_1 - V_{12})^2 + p_1 = \tfrac{1}{2}\rho(-V_{12})^2 + p_2. \tag{S12b}$$

Eqs. (S11) and (S12) are a system of four equations for the four unknowns u_1, V_{01}, V_{12} and h_2, while the parameters p_0 and h_0 are taken from clinical data, $p_1 = P + p_0$ is a prescribed input and h_1 and p_2 can be calculated using the constitutive law (S1) in terms of the other unknowns and problem parameters. We are primarily interested in calculating the pressure amplification at the sclera $R \equiv (p_2 - p_0)/(p_1 - p_0)$. To this end, equations (S11-S12) can be rearranged to give the amplification factor

$$R \equiv \frac{p_2 - p_1}{p_1 - p_0} = \frac{(h_1 - h_0)(h_2 + h_1)}{(h_1 + h_0)(h_2 - h_1)}. \tag{S13}$$

When combined with the constitutive law in Eq. (S1), Eq. (S13) is an implicit equation for the unknown h_2, which we solve using the fzero function in Matlab to yield the pressure amplification R. The amplification is plotted as a function of driving pressure in Fig. S10 (below), where we observe good quantitative agreement with the numerical simulations using pressure perturbations of finite duration for $T \lesssim 0.01$s. In particular, the curve overlaps closely with the profile for $T = 0.01$s and is identical in shape to the profile for $T = 0.005$s although offset in magnitude, both discussed below.

In using Eq. (S13) for the pressure amplification at the sclera, we have neglected interactions between the shock wave and rarefaction behind it, which may play a role if the input pressure pulse is of finite duration. This assumption can be justified by considering that a rarefaction wave at the back of a square input pressure pulse with finite interval T will be expected to travel with speed $u_1 + c_1$, where c_1 can be calculated from Eq. (S7). We find that the range of pressure pulses considered here would require approximately $T < 0.002$s for the trailing rarefaction wave to catch up with the shock before it hits the sclera, which is shorter than the input durations we consider in the numerical simulations below.

4 Numerical simulations

4.1 Numerical simulations without membrane tension

To simulate the model (Eqs. (S1), (S4)) in the absence of membrane tension across the domain ($0 \le x \le sL$) we use a sophisticated numerical method originally developed to study flow in the giraffe jugular vein [5]. This approach is based on a finite-volume method and allows for explicit shock capture. In this upwind numerical method we must also impose the fluid velocity at the site of perturbation ($\overline{u}(sL, t)$), but this must be chosen in a manner consistent with the downstream boundary condition (S4d) *i.e.* the fluid velocity ahead of the shock wave must remain undisturbed initially to satisfy the condition of no flow through the sclera. We construct the backward propagating characteristic which passes through $x = sL$ at that time. We then evaluate the Riemann invariant along this characteristic by projecting backwards in time to the previous timestep where the fluid velocity and pressure are known, and then use this invariant to compute the fluid velocity at the current step (a similar method was used by Sherwin *et al.* [6]).

4.2 Numerical simulations with membrane tension

To simulate the model with membrane tension (Eqs. (S4), (S8) and (S10)) across the domain ($0 \le x \le L$) we use a semi-implicit finite difference method adapted from Stewart *et al.* [9] which employs a first-order finite-difference stencil in time and a fourth-order finite-difference stencil in space. Despite the numerical method being semi-implicit, typical simulations require a large number of space and time discretisation points. For very short perturbation timescales ($T < 0.01$s) the system requires in excess of 10,000 spatial grid points across the domain and an extremely small timestep to accurately resolve the dispersive waves which appear ahead of the wavefront.

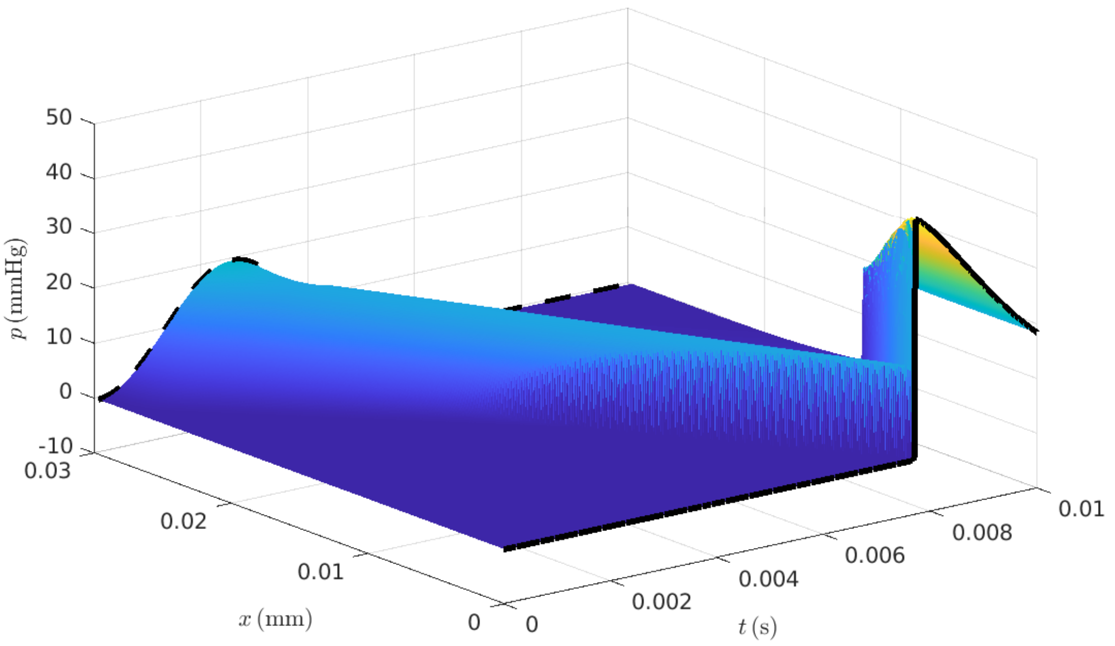

Figure S2: Carpet plot of the CSF pressure as a function of distance along the optic nerve and time in a simulation without membrane tension for perturbation timescale $T = 0.005$s and $P =$20mmHg.

5 Results and discussion

5.1 Typical results without membrane tension

Predictions of the model without membrane tension are described in Figs. 2-4 in the main text. To visualise the dynamics in an alternative manner, we further illustrate three-dimensional carpet plots of the CSF pressure as a function of both space and time.

A carpet plot of the CSF pressure for $T = 0.005s$ (complementary to Fig. 2 in the main text) is shown in Fig. S2, where the the inlet pressure perturbation triggers a propagating pressure wave which steepens to form a shock; arrival of this shock wave at the sclera drives an abrupt increase in CSF pressure.

A carpet plot of the CSF pressure for $T = 0.02s$ (complementary to Fig. 3c in the main text) is shown in Fig. S3, where the inlet pressure perturbation initially drives a smooth pressure increase at the sclera. This smooth pressure wave is reflected back toward the optic foramen, and subsequently steepens to form a shock wave. This shock wave is re-reflected back towards the sclera by the optic foramen, and its arrival at the sclera leads to a secondary (abrupt) increase in CSF pressure which exceeds the initial maximal pressure increase.

A carpet plot of the CSF pressure for $T = 0.05s$ (complementary to Fig. 3d in the main text) is shown in Fig. S4, where the inlet pressure perturbation drives a smooth pressure wave which propagates back and forth along the ONSAS; the resulting pressure trace at the sclera exhibits two local maxima over the timescale of perturbation.

In addition, we use the model without membrane tension to investigate three other features of the system with strong application to non-accidental head injury in infants, including an oscillatory inlet pressure mimicking a shaking event (Sec. 5.1.1), a persistently raised CSF pressure following a brain injury (Sec. 5.1.2) and a reduction in ONSAS length consistent with an infant (Sec. 5.1.3).

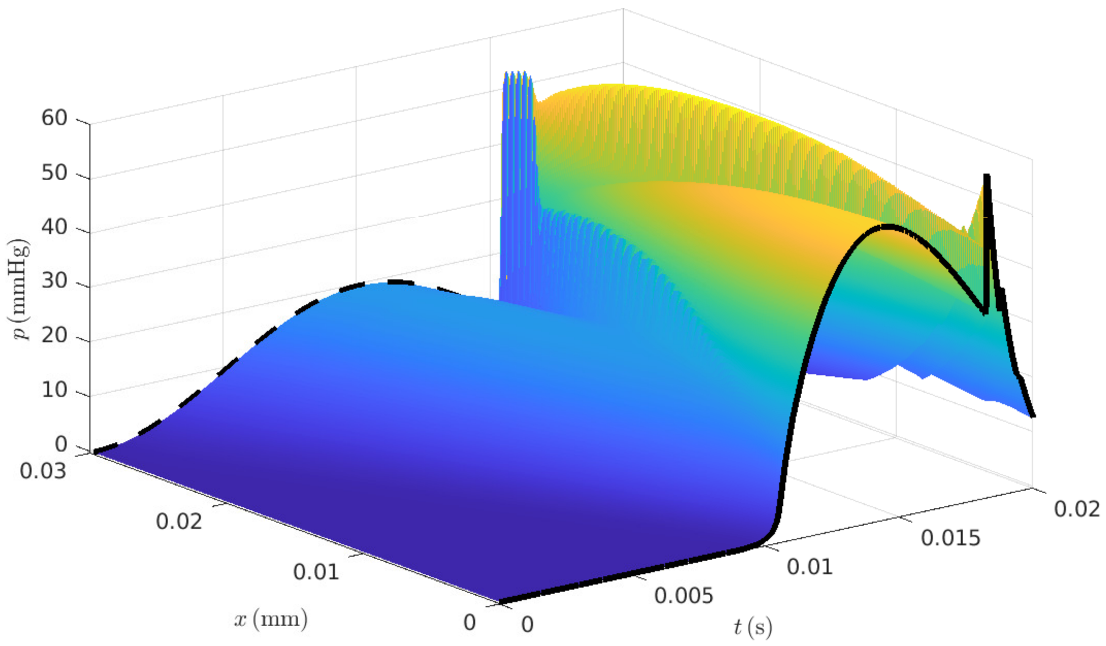

Figure S3: Carpet plot of the CSF pressure as a function of distance along the optic nerve and time in a simulation without membrane tension for perturbation timescale $T = 0.02$s and P =20mmHg.

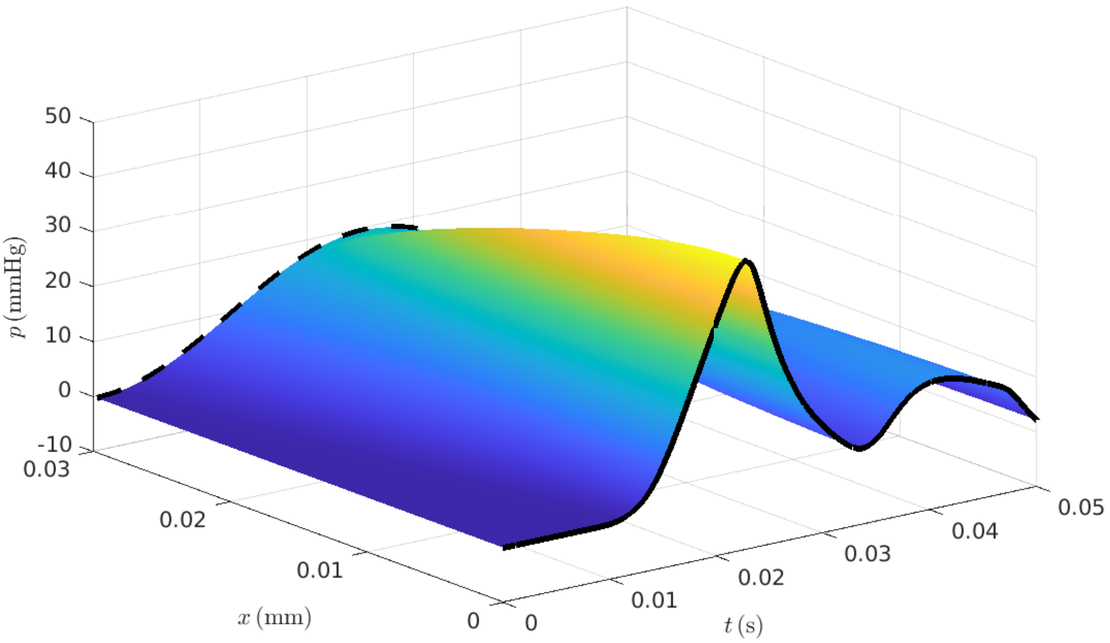

Figure S4: Carpet plot of the CSF pressure as a function of distance along the optic nerve and time in a simulation without membrane tension for perturbation timescale $T = 0.05$s and P =20mmHg.

5.1.1 An oscillatory inlet pressure

The simulations presented in the main text consider an isolated pressure increase and decrease over a fixed timescale. However, in some cases of traumatic brain injury, particularly inflicted head injuries caused by repetitive shaking, one might instead expect an oscillatory CSF pressure input.

In Fig. S5 we investigate examples involving an oscillatory input pressure with amplitude P and oscillation period T, where Eqs. (S4b,S4c) are replaced by

$$p(sL, t) = p_0 + \tfrac{1}{2}P\sin^2(\pi t/T), \qquad (t \geq 0). \tag{S14}$$

This oscillatory pressure forcing is plotted in Fig. S5(a,i) for $T = 0.005$s and $P = 15$mmHg, compared to the corresponding example with a single pressure increase/decrease at the inlet (Eqs. S4b,S4c). Time-traces of the corresponding scleral pressure are shown in Fig. S5(a,ii). As expected, these time-traces are identical for early times, exhibiting a first pressure amplification of ≈ 2.08 times the input at approx 0.008s, consistent with the analysis in the main text. However, beyond $t \gtrsim 0.015$s these two curves become distinct; both time-traces continue to oscillate, but the amplitude of the oscillation in scleral pressure generated by a single inlet pulse gradually decreases over time, while the amplitude of the oscillation generated by the oscillatory input is sustained over subsequent cycles and even magnified to ≈ 3.47 times the input pressure amplitude due to interaction with the propagation-reflection cycles of the original wave. Hence, in the latter case the global maximum scleral pressure is driven by secondary interactions between the propagating pressure waves, similar to the mechanism identified in the main text *i.e.* the oscillatory input pressure serves to amplify the scleral pressure through continued interactions between the waves propagating back and forth along the channel.

In order to determine how the period of this oscillatory input influences the pressure amplification at the sclera, Fig. S5(b) considers a fixed perturbation amplitude $P = 15$mmHg for several choices of oscillation period, plotting time-traces of both the input pressure (Fig. S5b,i) and the scleral pressure (Fig. S5b,ii). In each case the outcome is qualitatively similar to the case shown in Fig. S5(a): the resulting oscillations in scleral pressure do not settle into a fully periodic limit cycle (although the envelope of this limit cycle is approximately fixed), but instead the pressure waves continue to propagate back and forward along the ONSAS, where secondary amplification leads to a global maximum scleral pressure significantly greater than that due to the primary reflection. Across the three examples shown, the maximal scleral pressure amplification occurs for the intermediate case ($T = 0.01$): similar to the main text (and Sec. 5.1.1 above), this suggests an optimal perturbation timescale to achieve maximal pressure amplification arising due to secondary amplification of a propagating pressure wave.

5.1.2 A persistently raised inlet pressure

The simulations presented in the main text consider an isolated acute pressure increase and decrease over a fixed timescale T. However, in some cases of traumatic brain injury one might instead expect a persistently raised CSF pressure due to tissue oedema (swelling).

In Fig. S6 we investigate examples involving a persistently raised inlet pressure with amplitude P and ramping timescale $\frac{1}{2}T$, where Eqs. (S4b,S4c) are replaced by

$$p(sL, t) = p_0 + \tfrac{1}{2}P\sin^2(\pi t/T), \qquad (0 \leq t \leq \tfrac{1}{2}T), \tag{S15a}$$

$$p(sL, t) = p_0 + \tfrac{1}{2}P, \qquad (t \geq \tfrac{1}{2}T). \tag{S15b}$$

This persistently raised pressure forcing is plotted in Fig. S6(a,i) for $T = 0.005$s and $P = 15$mmHg (green solid line), compared to the corresponding example with a single pressure increase/decrease at the inlet (black dashed line) and for an oscillatory input pressure was the same properties (green dot-dashed line). Time-traces of the corresponding scleral pressure in these three cases are shown in Fig. S6(a,ii). As expected, all three traces are identical for early times, each exhibiting a first scleral pressure amplification at $t \approx 0.008$s, consistent with the analysis in the main text. However, while the scleral pressure subsequently decreases

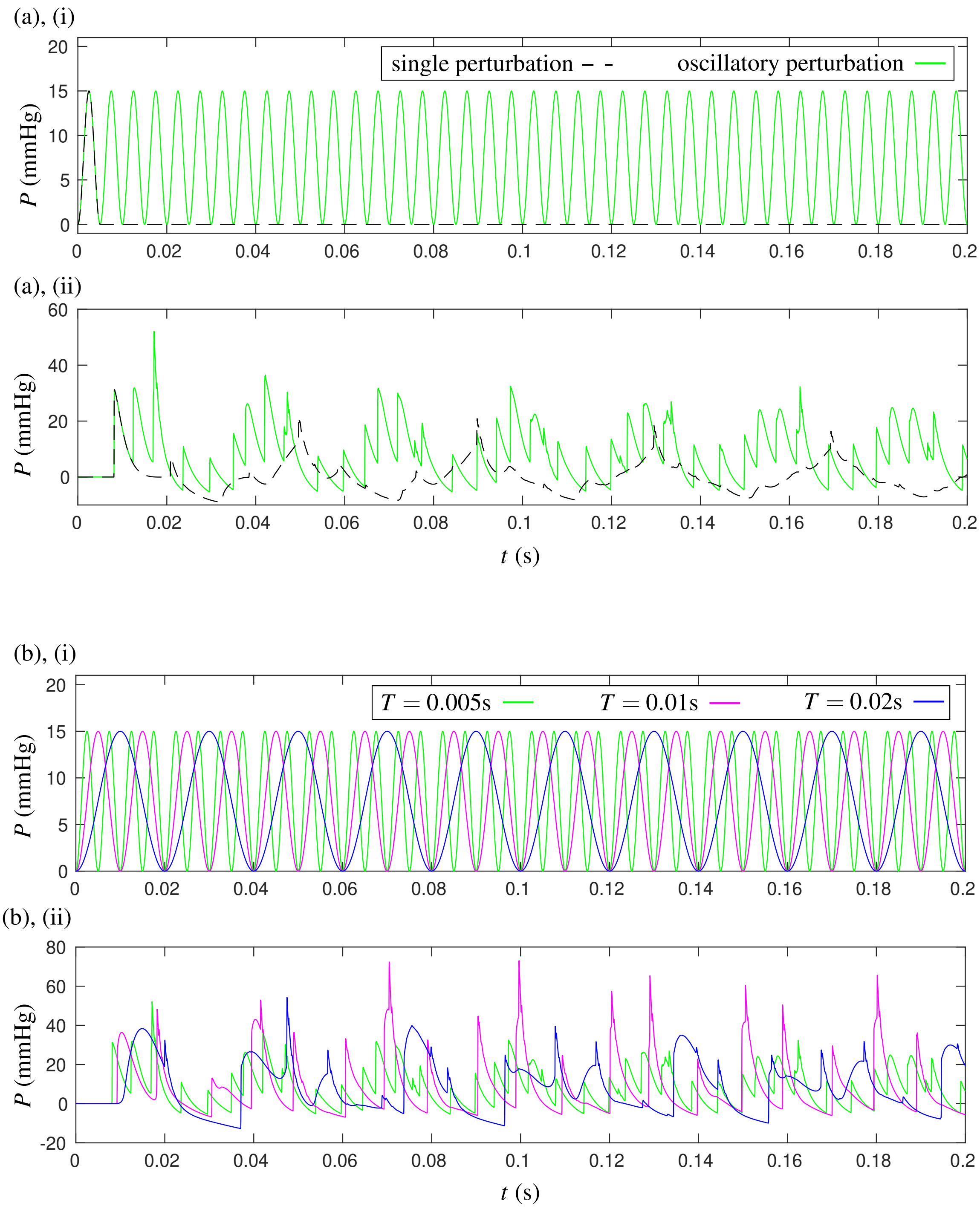

Figure S5: An oscillatory inlet pressure using the model without membrane tension: (a) time-trace of a single pressure perturbation (dashed line) compared to an oscillatory inlet pressure (solid line) for $T = 0.005$s; (b) comparison of the time-trace of the scleral pressure for three choices of the oscillation period $T = 0.005$s (green), $T = 0.01$s (magenta) and $T = 0.02$s (blue). Here $P =$15mmHg.

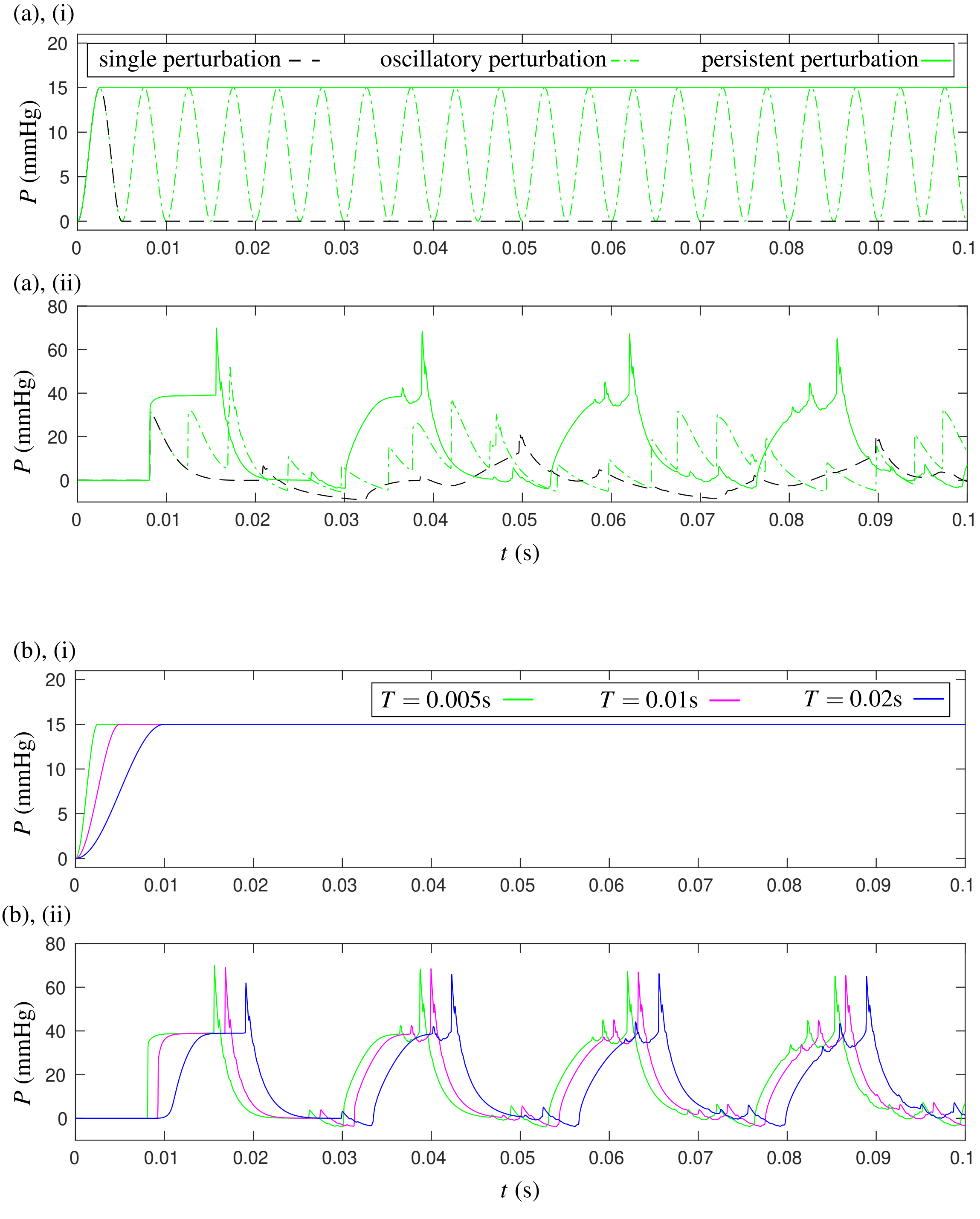

Figure S6: A persistently raised inlet pressure using the model without membrane tension: (a) time-trace of a single pressure perturbation (dashed line) compared to an oscillatory inlet pressure (solid line) for $T = 0.005$s; (b) comparison of the time-trace of the scleral pressure for three choices of the oscillation period $T = 0.005$s (green), $T = 0.01$s (magenta) and $T = 0.02$s (blue). Here P =15mmHg.

for both the single input pressure perturbation and the oscillatory pressure perturbation (dashed and dot-dashed lines in Fig. S6(a,ii)), the persistently raised input pressure (solid line in Fig. S6(a,ii)) transiently exhibits a plateau scleral pressure of $\approx$ 38.8mmHg before a secondary amplification increases the scleral pressure still further to a global maximum of $\approx$ 69.9mmHg; the mechanism of this secondary amplification is identical to that reported in the main text. Beyond this time the scleral pressure diminishes, and the system enters into a repeating (almost fully periodic) pattern with local maximal scleral pressures comparable to this initial maximum, although this local maximum does slowly decrease over time. Hence, the response to a persistently raised inlet pressure is qualitatively similar to a single input pressure perturbation, with propagating pressure waves moving back and forward along the ONSAS and the peak scleral pressure resulting from secondary amplification of these waves. However, this peak is larger than either the single input pressure perturbation or the oscillatory pressure perturbation as more energy is injected into the system.

In order to determine how the rate of inlet pressure increase influences the pressure amplification at the sclera, Fig. S6(b) considers a fixed perturbation amplitude P = 15mmHg for several choices of ramping time, plotting time-traces of both the input pressure (Fig. S6b,i) and the resulting scleral pressure (Fig. S6b,ii). In each case the outcome is qualitatively similar to the case shown in Fig. S6(a): the resulting oscillations in scleral pressure settle into a consistent (nearly periodic) pattern and the pressure waves continue to propagate back and forward along the ONSAS, where secondary amplification leads to a global maximum scleral pressure significantly greater than that due to the primary reflection. Across the three examples shown the maximal scleral pressure over each cycle is almost identical (beyond the first pressure amplification, where the ramping time does have a small effect) and there is only a short offset between the two (again due to the ramping profile). Hence, although the amplitude of the response is greater for a persistently raised ICP compared to a single inlet perturbation or an oscillatory input (for the same amplitude and ramping time), the mechanisms of pressure wave propagation and amplification remain identical to those reported in the main text. Note that the cases presented here are all for relatively fast ramping times. For much larger ramping times (where the CSF pressure rises slowly, such as in the presence of a tumour) the amplification in maximal CSF pressure at the sclera gradually reduces as T increases, and for large enough T we see no amplification at all.

5.1.3 A shorter ONSAS

The examples shown in the main paper all consider an adult ONSAS, where the length of the channel between the sclera and the OF is of length L = 27mm. However, since one of the primary applications of this work is to paediatric brain trauma, we further consider the same pressure amplification protocol in a shorter channel of length of L = 10mm, a more appropriate value to describe the ONSAS of an infant. It emerges that the dynamics are qualitatively (and almost quantitatively) similar to those reported in the main paper, where Fig. S7 illustrates panels analogous to Fig. 4 in the main text, summarising the type of wave which leads to the maximal pressure amplification (Fig. S7a) alongside contours of the corresponding maximal scleral pressure amplification (Fig. S7b). In each case the panels corresponding to a shorter ONSAS length are almost identical to those shown in the main text, with the exception that the optimal perturbation timescale which maximises the amplification at the sclera is slightly shorter and the corresponding scleral pressure amplification is very slightly reduced. In summary, this figure demonstrates that the mechanism of scleral pressure amplification described in the main text is unaffected by a shorter ONSAS length.

5.2 Typical numerical results with membrane tension

Inclusion of axial tension in the constitutive law (S8) prevents the dural profile from becoming infinitely steep and hence prevents formation of a shock wave. However, for short perturbation timescales the gradient of the dura mater can still become very large and approximate the formation of a shock. In order to compare to the simulations without axial tension, Fig. S8 revisits the baseline simulation from Fig. 2 in the main text,

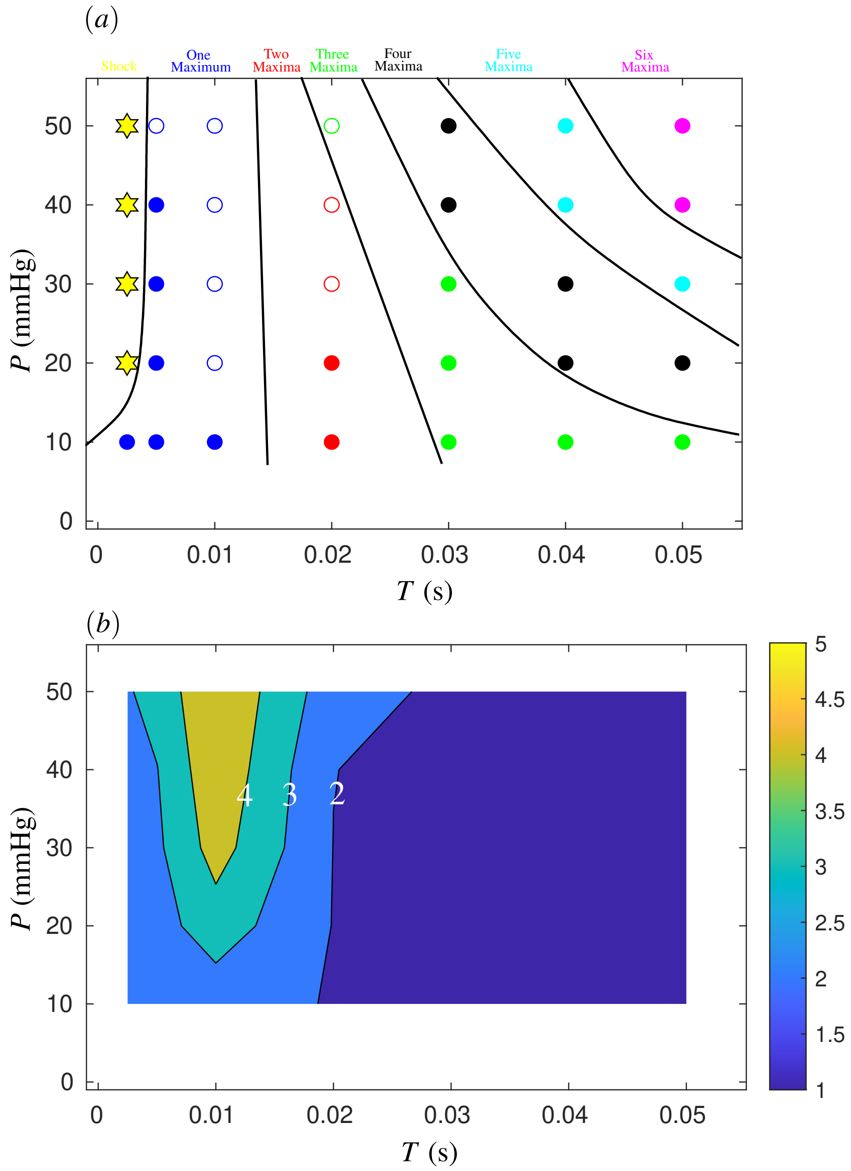

Figure S7: Influence of a shorter ONSAS length with $L = 0.01$m using the model without membrane tension. (a) Overview of the response to inlet pressure perturbation, where the maximal scleral wall pressure may be caused by the primary reflection of a shock wave (stars), propagation and reflection of a smooth pressure wave (filled circles) or as a result of secondary propagation/reflection of a shock wave combined with a smooth pressure wave (open circles). The number of maxima in the pressure profile at the scleral wall gradually increases with both the perturbation time and the amplitude of the pressure perturbation. (b) Color contour plot of the sclera pressure amplification, where the contour levels are measured relative to the amplitude of the maximal inlet pressure.

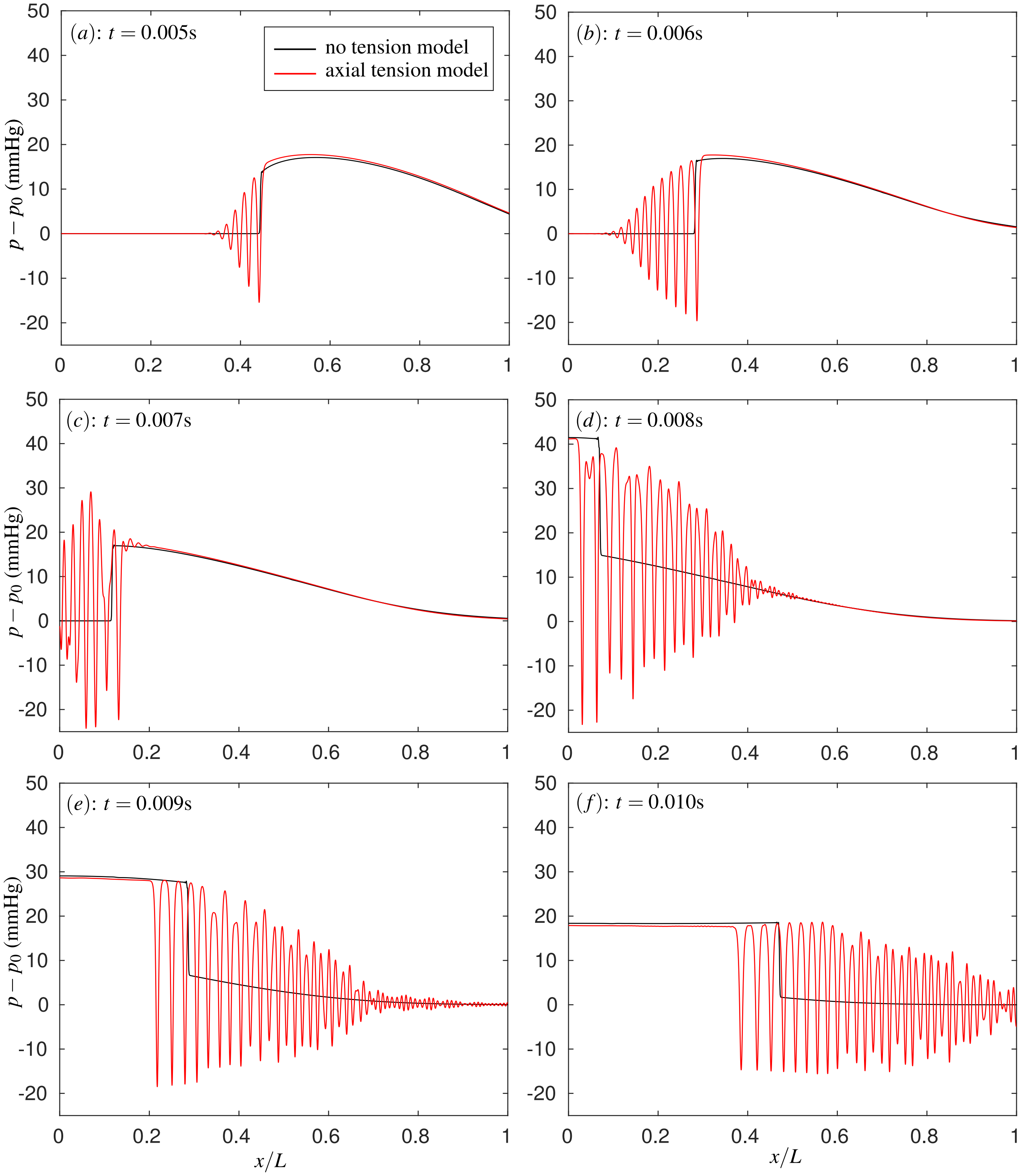

Figure S8: Snapshots of the spatial profile of the dura mater at six time points: (a) t =0.005s, (b) t =0.006s, (c) t =0.007s, (d) t =0.008s, (e) t =0.009s, (f) t =0.010s, comparing the profile from the shock capture method (solid black line) and the method with axial tension (solid red line). Other parameters are chosen as in Fig. 2 in the main text with $\mathcal{T} = 10^{-4}$.

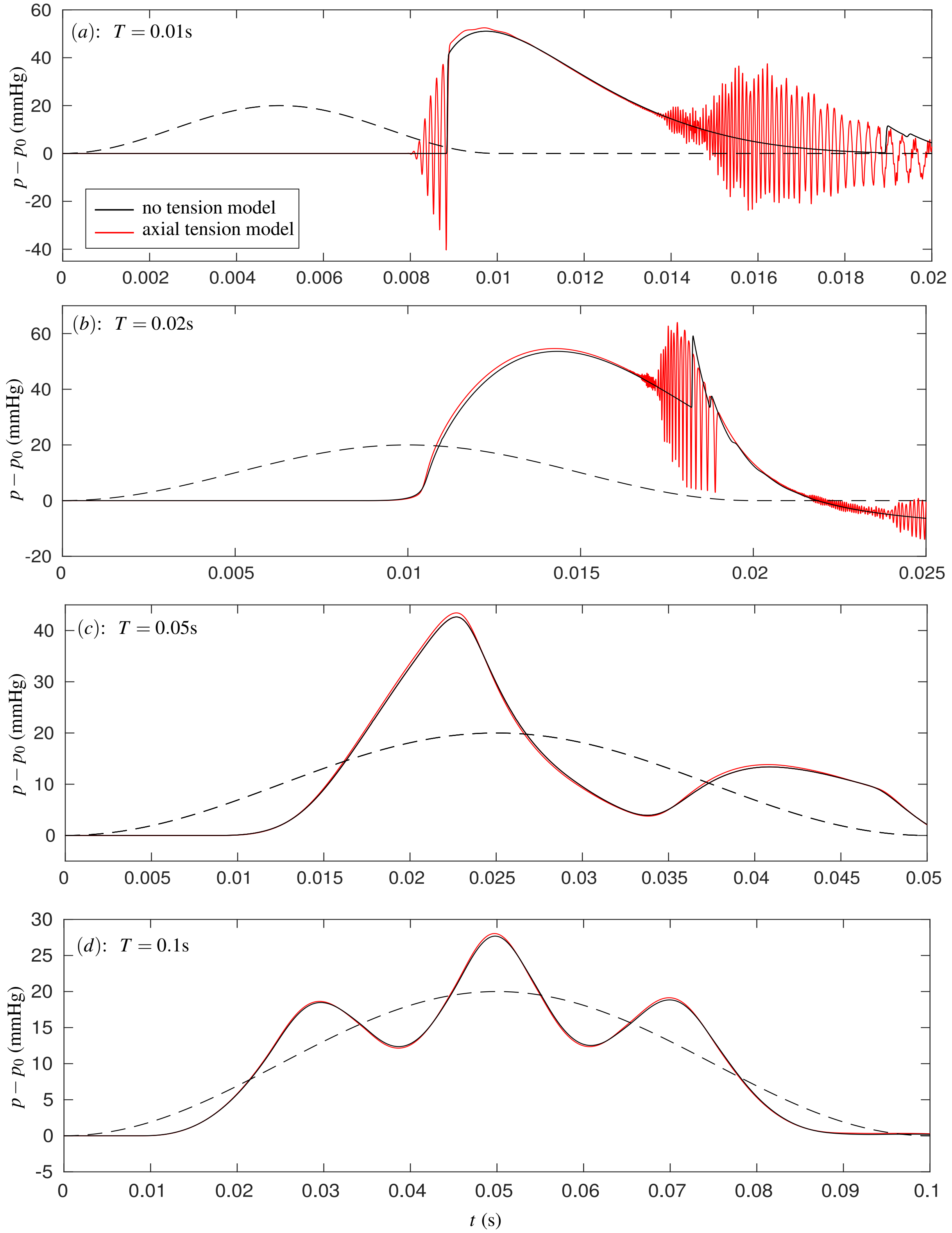

Figure S9: Time-traces of the CSF pressure at the proximal end of the optic foramen (dashed line) and the CSF pressure at the sclera in simulations without membrane tension (dot-dashed) and with membrane tension (red solid lines) for (a) $T = 0.01$s; (b) $T = 0.02$s; (c) $T = 0.05$s; (d) $T = 0.1$s. All other parameters are chosen as in Fig. 3 in the main text with $\mathcal{T} = 10^{-4}$.

superimposing the corresponding spatial profiles obtained with axial tension $\mathcal{T} = 10^{-4}$. Similar to the main text, the abrupt increase in driving pressure drives a rapidly propagating pressure wave along the ONSAS which becomes gradually steeper (Fig. S9a,b). The disturbance profile is quantitatively similar between the two approaches, with the exception that the inclusion of axial tension induces a region of dispersive waves ahead of the propagating wave front (decaying spatially in the direction of wave motion, with wavelength set by a balance between the axial tension and the elastic stiffness). For small values of axial tension, such as those considered here, these dispersive waves are extremely short wavelength and so require large numbers of spatial grid points to accurately resolve. However, despite this numerical difficulty we observe excellent qualitative agreement in the position and amplitude of the propagating wavefront across the panel where the pressure wave is advancing toward the sclera (Fig. S9a,b,c). As before, this propagating pressure wave is reflected as it encounters the sclera: the train of dispersive waves encounters the sclera ahead of the main wavefront, and these waves are sequentially reflected and superimposed on the portion of the wave-train still advancing towards the sclera; this superposition leads to a modest amplification in scleral pressure (Fig. S9c). However, a much greater amplification occurs once the main wavefront reaches the sclera, where the maximal pressure is almost identical to the prediction of the tension-free system (Fig. S9d). The reflected wave then propagates back toward the optic foramen with a train of dispersive waves ahead of the wave in the direction of propagation (Fig. S9e,f), although the reflected wavefront with axial tension now lags slightly behind the predictions of the shock capture system with no tension (Fig. S9e,f).

We note that these short-wavelength dispersive waves are a genuine prediction of the model with membrane tension and not a numerical artefact. In this study, we estimate tissue damage through the predicted local maximum in CSF pressure (which agrees very well between the two approaches), but note that our damage metric could be extended to include the effect of the large spatial gradients localised around the front of the wave. We previously investigated how the spatial decay rate these dispersive waves changes with the wall tension parameter in a related model of blood flow through the central retinal vessels ([10], see online supplementary material): the wavenumber of these dispersive waves is gradually reduces as the wall tension parameter increases, with their influence spread over increasingly long lengthscales around the wavefront. However, this smoothing of the profiles comes at a cost of reducing the overall wave amplitude, and for sufficiently large values of the wall tension parameter the localised wave propagation is completely suppressed. Investigation of this feature is deferred to future work, noting that there are currently no experimental estimates of the vessel wall tension *in vivo*.

To further examine the dynamics of the system with axial tension, Fig. S9 compares time-traces of the scleral pressure without axial tension (black solid line) to those with axial tension $\mathcal{T} = 10^{-4}$ (red solid line) for a variety of perturbation timescales with fixed perturbation amplitude $P = 20$mmHg; these examples correspond to time-traces in Fig. 3 in the main text. For very short perturbation timescales ($T \lesssim 0.01$s, Fig. S9(a)) the system exhibits an abrupt change in scleral pressure as the very steep pressure wave arrives (with a train of dispersive waves arriving shortly before), reminiscent of the arrival of the shock wave in Fig. 3b of the main text. As the perturbation timescale increases, the first increase in scleral pressure is more gradual (not associated with a shock wave), but, as in Fig. 3c of the main text, reflection of this smooth pressure wave results in a propagating pressure wave back towards the optic foramen; this reflected wave steepens until almost forming a shock (with a corresponding train of dispersive waves), which is then reflected by the optic foramen and generates a rapidly propagating shock wave back towards the sclera. Arrival of this secondary shock wave leads to a much greater maximal scleral pressure, similar to Fig. 3c in the main text but with a corresponding train of dispersive waves surrounding this maximum (see Fig. S9b for $T = 0.02$s). As the perturbation timescale increases further, the propagation and reflection of shock waves along the ONSAS becomes subdominant, and instead the system exhibits repeated propagation and reflection of smooth pressure waves with an increasing number of local maxima. For example, for $T = 0.05$s the system exhibits two local maxima over the perturbation timescale (Fig. S9(c), compared to Fig. 3d in the main text) while for $T = 0.1$s the system exhibits three local maxima over the perturbation timescale (Fig. S9(d), compared to Fig. 3e in the main text).

As a final comparison of the two approaches, in Fig. S10 we consider the amplification factor R defined

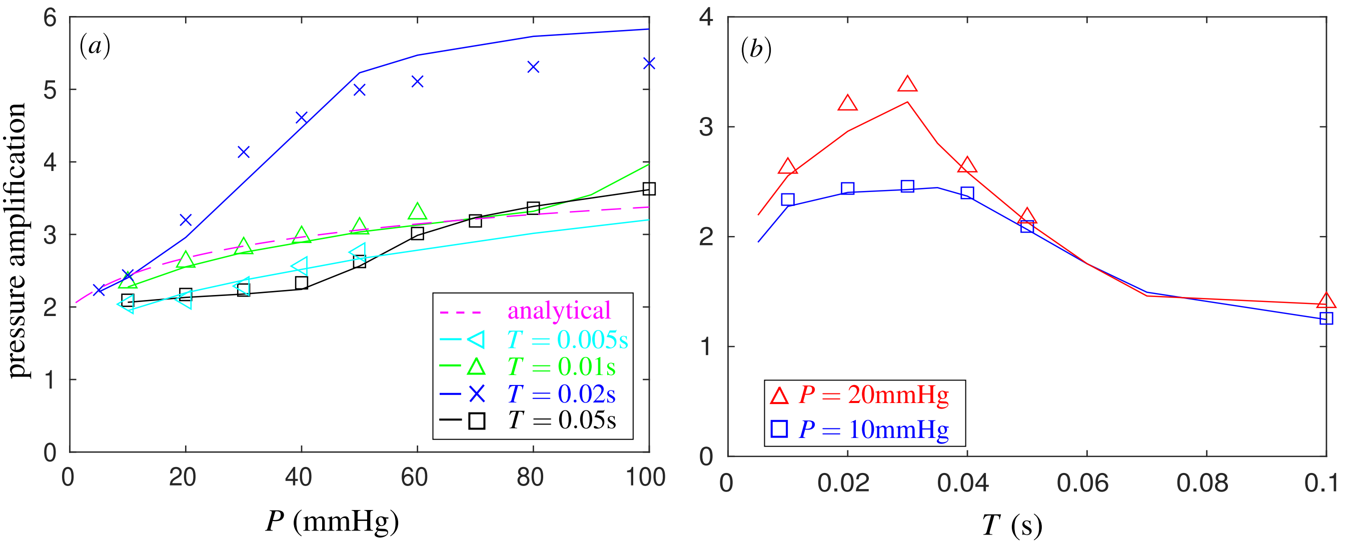

Figure S10: Maximal CSF pressure amplification at the sclera R defined in Eq. S5 measured as: (a) a function of perturbation pressure amplitude for fixed perturbation times $T = 0.005s$ (cyan) $T = 0.01$s (green), $T = 0.02$s (blue), $T = 0.05$s (black) (b) a function of perturbation time for fixed perturbation pressure amplitude $P = 10$mmHg (blue) and $P = 20$mmHg (red). All other parameters as in Fig. 3 of the main text. Open symbols in (a) and (b) are the corresponding predictions of the simulations with axial tension for the same model setup. The dashed magenta line in (a) is the corresponding prediction of the analytical model (S13).

in Eq. S5 plotted as a function of the both driving pressure P (Fig. S10a) and the perturbation timescale T (Fig. S10b). For short perturbation timescales (*e.g.* $T = 0.005$, $T = 0.01$), where the system admits a single propagating shock wave which is amplified as it is reflected at the sclera, the amplification factor gradually increases as a function of perturbation amplitude P (Fig. S10a), and the trend is well predicted by the simulations with no axial tension. Furthermore, for low perturbation timescales the analytical theory in Eq. (S13) is also in reasonable qualitative agreement. For larger perturbation timescales, where secondary propagation and reflection of a shock dominates the response, this amplification factor is much greater, with the maximal scleral pressure becoming more than five as the driving pressure amplitude, again showing strong agreement with the simulations with no axial tension (Fig. S10, Fig. 4 in the main text). For even larger perturbation timescales (*i.e.* $T \gtrsim 0.05$s) the predictions above indicate that the two methods are almost indistinguishable (Fig. S9), and this trend is also evident in the amplification factor, gradually increasing as a function of the driving pressure (Fig. S10a), but much less than the amplification driven by secondary shock propagation. If we instead hold the driving pressure amplitude fixed and vary the perturbation timescale, as expected we see a local maximum in the amplification factor when the response is dominated by the secondary propagation and reflection of a shock wave (*e.g.* $T \sim 0.02$s in Fig. S10b); this indicates that there is an optimal timescale for scleral CSF pressure amplification which is approximately independent of the driving pressure amplitude (also evident in Fig. 4b in the main text).

The strong quantitative agreement between these two numerical models (which are constructed and solved independently) gives us confidence in our numerical predictions. Despite the dispersive waves evident in the presence of axial tension, we observe excellent qualitative and quantitative agreement between the two numerical methods across the range of parameters considered. We now use this model with membrane tension to investigate the influence of optic canal tapering (Sec. 5.2.1).

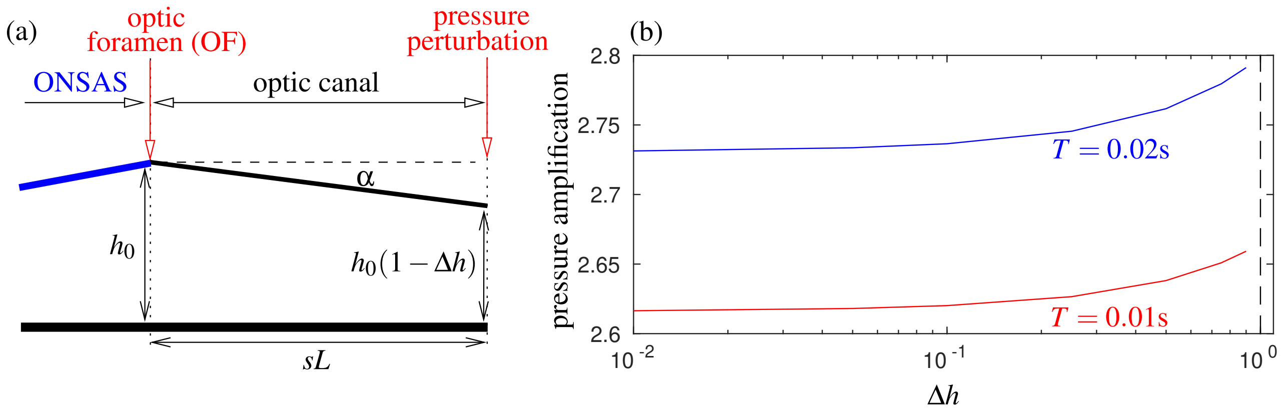

Figure S11: The influence of optic canal tapering computed using the model with membrane tension: (a) sketch of the tapered optic canal, illustrating the change in thickness along its length and the definition of the taper angle α and the taper parameter Δh; (b) corresponding predictions of the maximal scleral pressure amplification R measured as a function of the taper parameter Δh for both $T = 0.01$s (red) and $T = 0.02$ (blue). The black dashed line in (b) indicates complete occlusion of the canal. Here $P = 20$mmHg and all other parameters as in the main text.

5.2.1 ONSAS tapering

The model with membrane tension (Sec. 2.2) explicitly includes a segment of the ONSAS through the OF and along the optic canal, captured through boundary condition (S10); the derivation of this boundary condition assumes that the channel through the optic canal is of uniform width h_0. However, there is evidence to suggest that the optic canal tapers through the bone [11, 12], and so in order to mimic this effect we modify the model to assume that the channel walls of the optic canal are straight but no longer parallel, with the upper tapering at an angle α along the length sL; see sketch in Fig. S11(a), where we require $\tan\alpha < h_0/(sL)$ to prevent opposite wall contact along the length of the optic canal. In this setup the channel is of thickness of h_0 at the optic foramen and of thickness of $h_s = h_0(1-\Delta h)$ at the posterior end of the optic canal, where the taper parameter $\Delta h \equiv (sL/h_0)\tan\alpha$. Note that we need $\Delta h < 1$ to prevent opposite wall contact. The corresponding spatial profile along the optic canal can be written as

$$\breve{h}(x) = h_0\left(1 - \frac{\Delta h(x-L)}{(s-1)L}\right), \qquad (L \le x \le sL). \tag{S16}$$

The governing equations along this tapered channel region become

$$(\overline{u}\breve{h})_x = 0, \qquad \overline{u}_t + \overline{u}\overline{u}_x = -\frac{1}{\rho}p_x, \qquad (L \le x \le sL). \tag{S17}$$

We integrate the conservation of mass condition (the first equation of (S17)) to determine

$$\overline{u}(x,t) = \frac{\overline{Q}(t)}{\breve{h}(x)}, \tag{S18}$$

where $\overline{Q}(t) = \overline{u}(L,t)h_0$, the uniform flow rate along the tapered channel, is a function of t alone. Hence, the conservation of momentum equation (S17b) can be written as

$$\overline{Q}_t - \frac{\overline{Q}^2}{\breve{h}^2}\breve{h}_x = -\breve{h}\frac{1}{\rho}p_x. \tag{S19}$$

Since the tapered channel profile $\check{h}(x)$ is known explicitly through (S16), we integrate (S19) with respect to x to derive an expression for the pressure distribution along the tapered optic canal. We apply the same pressure forcing protocol as before, increasing the pressure at the distal end of the optic canal according to (S4b,c), providing the boundary condition needed to determine the constant of integration. Hence, the continuity of pressure boundary condition at the optic foramen takes the updated form

$$p(L,t) + \rho(s-1)L\overline{u}_t(L,t)\frac{\log(1-\Delta h)}{\Delta h} - \tfrac{1}{2}\rho\overline{u}^2(L,t)\left(\frac{1-(1-\Delta h)^2}{(1-\Delta h)^2}\right) = p(sL,t). \quad \text{(S20)}$$

Note that $\log(1-\Delta h)/\Delta h \to -1$ as $\Delta h \to 0$, and so boundary condition (S20) reduces to (S10) in the case of a uniform channel, as expected. Hence, tapering of the channel introduces additional inertia in the flow along the optic canal.

In order to determine the influence of optic canal tapering, Fig. S11(b) plots the maximal pressure amplification at the sclera as a function of Δh for two choices of the perturbation timescale ($T = 0.01$s, $T = 0.02$s) for fixed perturbation amplitude $P = 20$mmHg. In both cases we observe that the influence of changing the degree of channel tapering is minimal, and the system requires that the channel is almost completely closed at the distal end to see more than a 1% change in the maximal pressure amplification. In summary, this figure demonstrates that tapering along the optic canal makes almost negligible difference to the mechanism of pressure amplification identified in this study.

References

[1] Hansen HC, Lagreze W, Kruger O & Helmke K, Dependence of the optic nerve sheath diameter on acutely applied subarachnoid pressure – an experimental ultrasound study, *Acta Ophthalmol.* **89**, 528-532 (2011).

[2] Hansen HC & Helmke K, Validation of the optic nerve sheath response to changing cerebrospinal fluid pressure: ultrasound findings during intrathecal infusion tests, *J. Neurosurg.* **87**(1), 34-40 (1997).

[3] Stevens RF, Gommer ED, Aries MJH, Ertl M, Mess WH, Huberts W, Delhaas T, Optic nerve sheath diameter assessment by neurosonology: A review of methodologic discrepancies, *J. Neuroimaging* **31**, 814–825 (2021).

[4] Pedley TJ, The fluid mechanics of large blood vessels, Cambridge University Press (1980).

[5] Brook BS, Falle SAEG & Pedley TJ, Numerical solutions for unsteady gravity-driven flows in collapsible tubes: evolution and roll-wave instability of a steady state, *J. Fluid Mech.* **396**, 223-256 (1999).

[6] Sherwin SJ, Franke V, Peiró J & Parker K, One-dimensional modelling of a vascular network in space-time variables, *J. Engng. Math.* **47**, 217-250 (2003).

[7] Olufsen MS, Hill NA, Vaughan GDA, Sainsbury C & Johnson M, Rarefaction and blood pressure in systemic and pulmonary arteries, *J. Fluid Mech.* **705**, 280-305 (2012).

[8] Ockendon H & Ockendon JR, Waves and incompressible flow, Springer (2004).

[9] Stewart PS, Waters SL & Jensen OE, Local and global instabilities of flow in a flexible-walled channel, *Eur. J. Mech. B* **28**, 541-557 (2009).

[10] Spelman TA & Stewart PS, Shock wave propagation along the central retinal blood vessels, *Proc. Roy. Soc. A* **476**(2234), 20190269, (2020).

[11] Maniscalco JE & Habal MB, Microanatomy of the optic canal, *J. Neurosurg.* **48**(3), 402-406 (1978).

[12] Killer HE, Laeng HR, Flammer J & P Groscurth P, Architecture of arachnoid trabeculae, pillars, and septa in the subarachnoid space of the human optic nerve: anatomy and clinical considerations, *Br. J. Ophthalmol.* **87**, 777–781 (2003).
